# Harnessing BDNF Signaling to Promote Resilience in Aging

**DOI:** 10.14336/AD.2024.0961

**Published:** 2025-09-03

**Authors:** Jamshid Faraji, Gerlinde A. S. Metz

**Affiliations:** ^1^Canadian Centre for Behavioural Neuroscience, Department of Neuroscience, University of Lethbridge, Lethbridge, Alberta T1K 3M4, Canada.; ^2^Southern Alberta Genome Sciences Centre, University of Lethbridge, Lethbridge, Alberta T1K 3M4, Canada.

**Keywords:** Brain-derived Neurotrophic Factor, Brain, Aging, Resilience, Stress, Neurodegeneration, Neurodegenerative Disease, Alzheimer’s Disease, Parkinson’s Disease, COVID-19, Neurotrophins

## Abstract

As a key member of the neurotrophin family in the central nervous system, brain-derived neurotrophic factor (BDNF) plays a critical role in the maintenance and plasticity of the nervous system. Its innate neuroprotective advantage can also be shared with the brain when normal aging-dependent processes challenge neural circuits. The intricate relationship between BDNF and resilience during the aging process signifies the molecular mechanisms that underlie the maintenance and protection of brain function, such as cognition, movement and psychological well-being. As BDNF is crucial for neuronal growth and survival, it can also promote resilience against age-related functional decline and frailty, even if it fails to entirely prevent aging-related functional decline. In the present review, we discuss BDNF function from a neuroprotective perspective and how it may promote resilience in aging. We emphasize briefly the principal, well-known cellular hallmarks of brain aging and how BDNF may restrict such disabling molecular dynamics and enhance overall functional resilience in aging. Insight into the molecular pathways through which BDNF reduces age-related brain dysfunctions and/or improves resilience, provides a foundation for developing targeted interventions to promote mental well-being in an aging population.

“This small, thatched roof hath a mighty spirit dwelling within”. *Hamlet* by W. Shakespeare (1564-1616).

The global aging population (defined as those aged over 60 years) is rapidly growing [1, 2]. Given the influence of unfavorable cultural and lifestyle factors (e.g. stress, poor social engineering, malnutrition) as well as adverse environmental conditions such as climate change, pandemics and social conflicts, extended longevity may not necessarily be linked to a prolonged period of good health and resilience. All these concerns represent just a snapshot of the multifaceted complications occurring within the field of aging research, which involves multiple disciplines ranging from social engineering and public health to psychology and sociology. For example, with the increasing frequency and intensity of natural disasters due to climate change, attention to resilience building strategies is necessary to effectively mitigate the impacts of extreme weather events in aged individuals. Moreover, understanding how aged populations can recover from adversity, whether it is natural disaster, economic instability, or social conflict, is essential for fostering resilience at the grassroots level. These challenges explain why the UN declared 2021-30 as the *Decade of Healthy Aging*. Nevertheless, beyond the empirical level, it is essential to provide a conceptual framework for the dynamic processes of aging that enables actionable decision making for future interventions [3]. Whether aging is accelerated by damage accumulation over time, caused by disrupted homeostasis or linked to adverse developmental trajectories, there is still a chance that most interests in the field converge to a point of consensus: functional decline and deterioration that may compromise resilience. The brain, similar to various physiological systems, undergoes a gradual decline in functional capacities throughout the aging process, leading to impairments in cognitive domains such as learning, memory, attention, decision-making, sensory processing, and motor coordination. These manifestations are indicative of the progressive functional deterioration associated with aging [4]. For the present discussion, a time-dependent functional decline that is typically associated with progressive deterioration of physiological and cellular integrity may explain the aging process in different biological systems including the brain. More specifically, the normal aging process in the brain is accompanied by gradual molecular downregulation and cellular degradation, leading to cerebral atrophy and ultimately age-related brain dysfunction [5, 6].

Notably, aging is associated with a higher risk of neurodegenerative, inflammatory, and metabolic pathologies, which raises several intertwined conclusions. First, the impact of the aging process manifests heterogeneously among organ systems and individuals [*mosaic aging* [7]]. However, despite this variability, several shared mechanisms known as *hallmarks* [8] or *pillars* [9] of biological aging contribute to neuronal dysfunction in the elderly population. Second, the overt manifestations of brain aging are not uniformly exhibited across all brain structures. The specificity of these regional responses suggests selective vulnerabilities of certain functional networks to brain aging. For example, the hippocampus [10, 11] and cortical somatosensory and somatomotor areas [12] appear more vulnerable to neuronal aging than others. The molecular and cellular changes that occur during aging in these structures over time usually result in a poor functional profile, primarily in cognition (e.g. impaired spatial learning and memory) and movement (e.g. frailty and/or loss of balance). The region-specific aging of the brain along with physiological changes may also contribute to reduced resilience and lower adaptation to life adversities. Third, when people get older, the segregation of brain systems or specialization in brain function progressively decreases. The functional dedifferentiation in the aging brain ranges from the firing patterns of single neurons to the evoked activity of individual brain regions [13]. Interestingly, higher segregation of the brain’s connectome into distinct functional networks serves as a modulating factor of cognitive decline and resilience in aging-related degenerative conditions [14]. Lastly, while aging is known to progressively contribute to disease and poor resilience, a reciprocal relationship between aging and disease can also occur; diseases and/or their treatments might also expedite the development of aging-associated pathologies [9]. This is specifically important when one is concerned with understanding the aging process to recommend pathways as therapeutic targets or for preventative interventions, or even to employ this knowledge to extend healthspan in later life.

Chronological changes are not directly equivalent to the rate of biological alterations over time [15]. Thus, prior to any further discussion on how aging-related processes challenge overall resilience, it is necessary to distinguish between chronological and biological aging. While chronological aging refers to the actual number of years a person has been alive from the date of birth, biological aging (also known as physiological or functional aging), denotes the condition of a person’s body and its systems relative to their chronological age. Accordingly, biological aging reflects how well or poorly an individual’s body is functioning compared to the average person of the same chronological age. Biological aging is closely linked to experience-dependent patterns of epigenetic regulation of gene expression [16]. Undoubtedly, biological aging, as opposed to chronological aging, is correlated with substantial alterations in many hallmarks of health [17]. These changes reflect progressive deterioration of tissues and biological systems associated with the progressive senescence of organellar, cellular, organismic, and systemic functions. An optimal response to life-threatening factors in aging, therefore, is critically linked to the overall resilience that may be directly impacted by these pervasive incapabilities. Additionally, reduced resilience in aging may in turn accelerate aging-dependent complications. This process may call for multi-level (systemic or lifestyle) therapeutic interventions that can properly address a set of influential factor (s) and their correlates. Further, the rate of biological aging typically varies among different tissues and systems within the body. However, compared to other systems, brain aging follows an entirely unique pattern of alterations, enabling it to profoundly affect the biological aging process in other body systems. This may occur through various interconnected pathways such as the autonomic nervous system, the hypothalamic-pituitary-adrenal (HPA) axis, and the endocrine system such as the hypothalamic-pituitary-gonadal (HPG) axis. However, the extent to which brain aging can influence or predict aging processes in other systems is still an area of ongoing research. Though controversial [18], neuroimaging measures indicate that brain age predicts mortality [19], and brain aging in return is reciprocally impacted by the biological aging seen in other organ systems [20]. For example, because the gut microbiome and its composition changes with age, the microbiome may contribute to the link between the aging gut and the aging brain [21, 22]. Thus, the brain’s biological aging may predict the biological aging processes of other body systems [15].

It is promising to note that brain aging-dependent cellular impairments, which nearly consistently imply cellular senescence, and their subsequent cognitive decline can be restored to more youthful levels [5]. Therefore, any intervention that builds resilience (e.g. cognitive reserve) in the older population appears more constructive when it directly addresses specific brain functions at different levels of manipulation. Indeed, factors such as genetics, lifestyle, environmental influences, and overall health status can also a play significant role in shaping the aging trajectory of different body systems. Hence, although the brain may contribute to the regulation and coordination of aging processes throughout the body, its ability to precisely predict these processes may be influenced by a complex interplay of multiple factors.

**BOX 1: Overview:** *Resilience as a Process and its Practical Application to Aging* Resilience as a dynamic and multifaceted process involves the ability of organisms to recover from adversity, cope with stress, and adapt to challenges. The correlates of resilience as a process include: *(i) Exposure to adversity or challenge. Resilience* typically begins with the encounter of adversity, stress, or a significant life challenge. The nature and severity of the challenge can vary widely between individuals. Notably, individual differences determine heterogeneity of responses to challenges. In a practical approach, it is important to recognize that aging brings about various challenges, including physical, cognitive, emotional, and social changes. Practitioners should encourage their clients to embrace these challenges as opportunities for growth and learning rather than insurmountable obstacles. *(ii) Initial response*. When faced with adversity, individuals, including the elderly experience an initial reaction that may involve emotions such as frustration, fear, sadness, or anger. This initial response is a natural part of the process and can vary depending on factors such as personality, past experiences and learning history, and the specific nature of the challenge (see BOX 2). In a practical approach, it is critical to acknowledge and validate the emotions that arise in response to aging-related challenges. Practitioners should encourage their clients to practice self-compassion and remind themselves that it is normal to experience a range of emotions when facing transitions in life. *(iii) Coping mechanisms*. Resilience involves the use of coping mechanisms to manage the stress and emotions associated with adversity. Coping strategies can be both problem-focused (actively addressing the source of stress) and emotion-focused (regulating emotional responses to stress). Two fundamental psychological aspects of resilient behaviours are self-regulation and locus of control [26] which dictate both the direction and endurance of coping mechanisms. In a practical approach, it is important to develop adaptive coping strategies to effectively manage stress and navigate through aging-related challenges. This strategy may include seeking social support from friends and family, engaging in regular physical activity, and participating in social activities to find meaning and purpose in daily life. *(iv) Adaptation and adjustment.* As individuals navigate through adversity and apply coping mechanisms, they gradually adapt to the new circumstances and adjust their thoughts, behaviours, and emotions. This may involve developing new skills, changing perspectives, or finding alternative ways to meet demands, goals, and needs. In a practical approach, it is important to remind clients to remain flexible and open to adapting to changes in physical health, cognitive function, and lifestyle preferences as they age. Practitioners should encourage their clients to focus on what they can control and inspire creative solutions to overcome obstacles and maintain independence and autonomy. *(v) Learning and growth*. Resilience often leads to personal development. Through the process of overcoming adversity, individuals may gain new insights about themselves, build self-confidence, strengthen coping skills, and develop a deeper sense of purpose or meaning in life. These experiences can contribute to increased resilience in the face of future challenges. In a practical approach, it is critical to take advantage of opportunities for personal growth and development as individuals age. Practitioners may encourage clients to stay curious and engaged in lifelong learning activities, such as learning new skills or pursuing hobbies and interests that bring them joy and fulfillment. *(vi) Integration and maintenance*. Finally, resilience is an ongoing process that involves integrating the lessons learned from past experiences and maintaining adaptive coping strategies over time. This may even include achieving post-traumatic growth, which is a key component of resilience, as people transform distressing experiences into opportunities for personal development and deeper understanding [270]. Individuals who have developed resilience are better equipped to navigate future challenges, as they have built a repertoire of effective coping skills and have confidence in their ability to overcome adversity (Fig. 1). In a practical approach, it is important to encourage clients to integrate resilience-building practices into their daily routines to maintain emotional wellbeing and cope with the fluctuations of aging-related experiences. This may include practicing gratitude, staying connected with supportive social networks, prioritizing self-care activities, and maintaining a positive outlook on life. Social support also has a central role in building resilience [42]. Hence, it is important to spend quality time with family and friends and seek out meaningful community engagement.

## Resilince, natural acumen of the organism

While resilience has recently been introduced into the natural science discourse, it is not a novel concept. Derived from the Latin root "resilire," meaning to spring back (or the ability to rebound), resilience was initially employed by physical scientists to signify the characteristics of a spring and explain the stability of materials and their resistance to external shocks. The contemporary psychobiological formulations of resilience uphold the core idea that there can always be a strategy to promote fortitude or resilience even in highly vulnerable biological systems. Thus, resilience from a natural science viewpoint is an end-product (not a dead end) of a counter-regulatory process that may modulate a system towards a more functional condition when challenged [[23], see also [24] for further discussion]. Conceptually, resilience is not necessarily about the absence of vulnerability, however [25]. Instead, it refers to a dynamic process of adaptation (BOX 1) to adversity while a consistent, functional homeostasis or the maximum biobehavioural preparation is maintained. Even if we formulate resilience as a *capacity* or an *ability* [26, 27], the fact hidden behind it is a potentially well-orchestrated, productive dynamic that may support the process of adaptability to achieve a successful outcome in the face of adversity or stress. In the present discussion, therefore, we suggest that resilience is a condition that involves dynamic developmental, genetic, epigenetic, and neurochemical processes for optimizing adaptive biobehavioural responses to stressors or aversive experiences.

**Figure 1.**
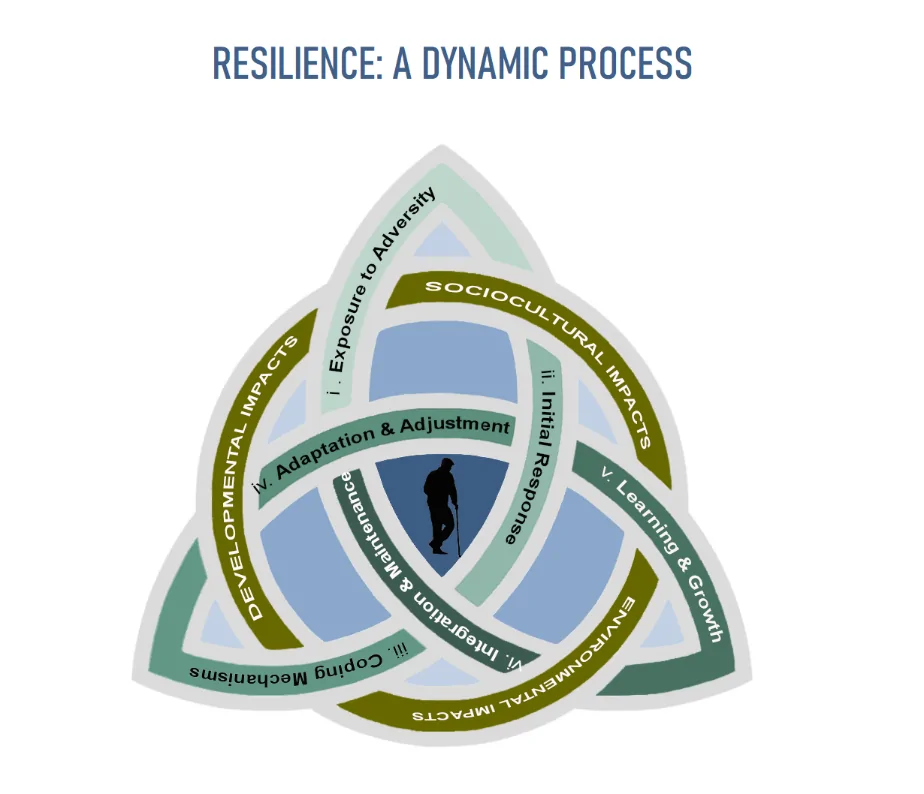
**Resilience as a process**. Resilience is often described as the capacity to recover quickly from difficulties (e.g., developmental and environmental impacts); however, understanding resilience as a process rather than a static trait or outcome offers a deeper and more dynamic perspective. Viewing resilience as a dynamic process offers a nuanced and holistic understanding of how individuals navigate adversity. It highlights the dynamic interplay between personal attributes and external resources/imapcts, emphasizing growth, adaptation, and continuous development.

This viewpoint requires that resilience modalities be expanded from a mere biobehavioural domain to also consider the psychology of resilience [28] where the stability or disruption in psychological functioning is addressed (BOX 2). However, when *development* becomes a factor in the equation [29], a practical approach is also encouraged to promote lifetime health trajectories. For example, development appears to influence the heterogeneity of responses to adversity throughout a lifetime through an interplay with environmental or contextual factors, such as lifestyle. While some individuals may wilt under pressure, others may bloom and thrive, exhibiting empowerment over environmental susceptibility. Hence, slightly- and highly-reactive phenotypes do not simply represent two alternative states of calmness and overarousal in neurobiological pathways. Instead, they refer to sensitivity to both protective and harmful contextual effects, known as the developmental dichotomy of *orchid* versus *dandelion* metaphor of resilience [30]. This metaphor refers to two distinctive patterns of responses in which orchids represent those individuals who do exceptionally well in ideal conditions but who also respond more strongly to poor conditions. By contrast, dandelions are less sensitive to environmental quality, i.e. they are more resilient, adapt readily to environmental changes and thrive [30]. In the context of aging, where it is seen in the continuum of development and as an environmental susceptibility, not only the biobehavioural resilience, but other concerns arise about psychological resilience, such as cognitive aging and mental health in the elderly. These include, for instance, how older individuals cope with loss (e.g. loss of independence), the strategies by which they adapt to health changes (e.g. cognitive and motor decline), how they face existential challenges (e.g. sense of acceptance and gratitude), and more importantly, how to promote resilience in the elderly in this context. Hence, regardless of how elderly individuals respond to adversities, and how varied these responses are, the existing conceptual frameworks of resilience are expected to identify a way to how resilience can be built and optimized.

**BOX 2: Outlook:** *From Conflict to Growth* Resilience, the dynamic process of adaptation to adversity, represents two major intertwined responses (*1*) *during* a challenge, and (*2*) when the challenge is *over*. Resilience during a challenge (*resilience in a confrontation*) refers to the ability to remain composed, adaptable, and effective when facing challenging or adversarial situations. This involves maintaining mental strength, emotional stability, and strategic thinking during conflict or confrontation. As confrontation deepens, the resilience process requires an organism to be more purposeful and goal-directed until the challenge is terminated. Resilient individuals, therefore, can withstand pressure, criticism, or hostility without losing their sense of self-control or purpose. They also tend to approach stressful situations with a sense of agency, actively seeking solutions and taking steps to manage and cope with difficulties. This sense of control empowers them to take effective action and maintain a sense of mastery over their circumstances. Such individuals are capable of bouncing back from setbacks, learning from experience, and even using confrontation as an opportunity for growth or positive change. The ability to express personal thoughts, feelings, and needs confidently and respectfully (assertiveness) and maintaining personal perspective during confrontations are the most representative aspects of psychological resilience. Taken together, resilience in a confrontation is about facing difficult circumstances and stressful situations with courage, commitment, control, and a constructive mindset. Resilience when a challenge is over (*resilience in restoration*) implies the ability of individuals to recover, adapt, and thrive in the face of adversities or stressors. This response can also be applied within a broader framework when the ability of ecosystems, habitats, or communities is addressed to recover and persist following adversities, disturbances, or environmental stresses. However, if it is applied in the context of psychobiological restoration, the response entails the capacity of an organism to be fully restored after being disturbed. The process pertains to the capacity of the organism to recover and regain its optimal functionality (or the maximum biobehavioural preparation) following disturbances. It encompasses factors such as physiological function, organismal homeostasis, psychophysiological integrity, and behavioural stability. Further, resilience in restoration can extend to social and economic aspects of adaptation, focusing on the ability of individuals to recover and rebuild in the aftermath of interpersonal crises or disruptions, such as social conflicts and rejections as well as economic downturns. Overall, resilience in restoration emphasizes the importance of building and maintaining systems that can survive and recover from adversities, trauma, and stresses while sustaining their essential functions and services. During restorative response, resilient people also exhibit stability in the face of new disturbances up to a certain threshold. Beyond this threshold, they may undergo abrupt changes or collapse into an alternative state, leading them to suboptimal or nonoptimal resilience.

Resilience needs to be considered in light of the degree or dose of exposure to adversities over time [23, 29, 31], whether it is a single highly traumatic incident or cumulative effects of repeated on enduring stressors. Both short-term traumatic experiences and persistent stress appear to significantly influence physiology and health outcomes, especially in aging when most coping systems are more vulnerable and at higher risk of failure. Chronic stressful experiences in older adults may contribute to accelerating immune aging by decreasing naïve and increasing terminally differentiated T-lymphocyte percentages [32]. Thus, the accumulation of adversities and stressful events across the life course has a significant impact on age-related activation of the immune system. At a mechanistic level, however, reduced immunological resilience to stress in the elderly is entangled with a common point of convergence across various biological systems that fail to optimally function in the face of stress or have difficulty to recover from adversity.

A conceptual framework that considers aging of multiple systems linked to cumulative lifetime stresses is allostatic load [33]. The study of allostatic load has become valuable in providing sets of biomarkers that assess multi-system “wear and tear” and pathological risk over time - allostatic load increases as the body attempts to cope with stressors, and a high allostatic load predicts a higher risk of aging-associated diseases. Various types of allostatic load indices with measurements from the endocrine, immune, metabolic, and genomic domains have been adopted by clinical studies [34-36] but lately also introduced to animal models to facilitate knowledge translation [37, 38]. These approaches, together with epigenetic aging clocks [39-41], have become robust tools in assessing biological aging and prognosticating future health- and lifespan trajectories.

## Resilience from a developmental and immunological perspective

Mere biological approaches to resilience fail to fully explain sociocultural and environmental impacts if developmental inputs are underestimated. The developmental dynamics, in turn, reflect the impact of social interactions and environmental influences on overall biological resilience [23, 42]. Resilience in aging also follows a similar trajectory. That is, developmental components including social relationships, ecosystems, and organizations may shape the nature of the adaptive biological systems such as immune system function later in life. Developmental trajectories of resilience also involve time-dependent milestones and critical periods that along with sociocultural inputs may drive biological systems to either flexibility or vulnerability [43]. Perinatal influences can change physiological systems in children, potentially affecting childhood health and overall resilience [44]. For example, childhood self-control predicts physical health, and children with lower self-control have poorer biobehavioural outcomes [45].

In addition, aging is typically associated with profound physiological alterations that affect the immune system. The aging immune system is prone to immunosenescence, a condition that mainly involves profound changes in immune parameters such as a reduction in the number of peripheral blood naïve cells and a relative increase in the frequency of memory cells when compared to young healthy individuals [46, 47]. Cellular aging-induced deterioration of immune cells and programmed cell death (apoptosis), such as observed in thymus and spleen, are regulated by epigenetic processes [48]. Also, immunosenescence coupled with chronic low-grade inflammation in aging known as inflamm-aging [49, 50], is the underlying cause of many diseases and reduced resilience in older individuals. Therefore, the aging-related immune decline represents a normal gradual deterioration, profound remodeling, and significant changes in immune function with a profound impact on immune resilience [51]. It appears that an immunologically resilient phenotype (high immunocompetent) aligned with a specific immunocompetence-inflammation balance is linked to favorable immunity-dependent health outcomes [51]. Conversely, failure to preserve and/or restore optimal immune resilience when aged individuals experience immunoinflammatory stressors is a key to health-related challenges. Though developmentally dictated, immunosenescence has not yet been extensively explained in terms of its molecular and cellular foundations. Nevertheless, it is associated with the overproduction of proinflammatory cytokines such as interleukin 1β (IL-1β), interleukin 6 (IL-6), interleukin 18 (IL-18), C-reactive protein (CRP), and tumor necrosis factor alpha (TNF*a*) in the innate immune system [52-54]. In the central nervous system (CNS), these alterations may result in reduced levels of brain-derived neurotrophic factor (BDNF), leading to glutamatergic activation and consequently triggering three major pathways of neuronal death: excitotoxicity, oxidative stress, and the induction of apoptosis [6, 55, 56], all of which contribute to cognitive decline in the aging brain. For instance, BDNF levels predict the need for intensive care in elderly with COVID-19, suggesting that lower BDNF levels may serve as a biomarker for more severe disease progression and poorer clinical outcomes in an age- and sex-specific manner [57, 58], especially when infection leads to severe pro-inflammatory cytokine responses. It appears that older men respond to COVID-19 infection with lower concentrations of BDNF [57]. Thus, BDNF may be a critical determinant of severe COVID symptoms and a key component in long COVID syndrome [59].

Although this topic falls beyond the scope of the present review, it is worth noting that the immune system acts in close dialogue with the neuroendocrine system including the HPA axis, where its optimal resilience is regulated by the release of glucocorticoids (GC) [46, 60]. GCs normally inhibit the release of proinflammatory cytokines such as IL-6. However, when the HPA axis experiences chronic dysregulation influenced by persistent adversities, the ability to restrict proinflammatory cytokine production may diminish, leading to heightened inflammation and consequently suboptimal or even nonoptimal immune resilience [61]. Unsurprisingly, BDNF was shown to be highly involved in HPA axis regulation [62-64] indicating that it is a key player in the maintenance of homeostasis, one of the hallmarks of optimal resilience at different levels of cellular function. Prolonged HPA activity, on the other hand, may reduce BDNF signaling and its receptor TrkB in the CNS [65]. It appears that BDNF downregulation critically contributes to the loss of resilience in the elderly, although the primary causes that perturbate optimal resilience process in aging have not been fully elucidated yet.

**Figure 2.**
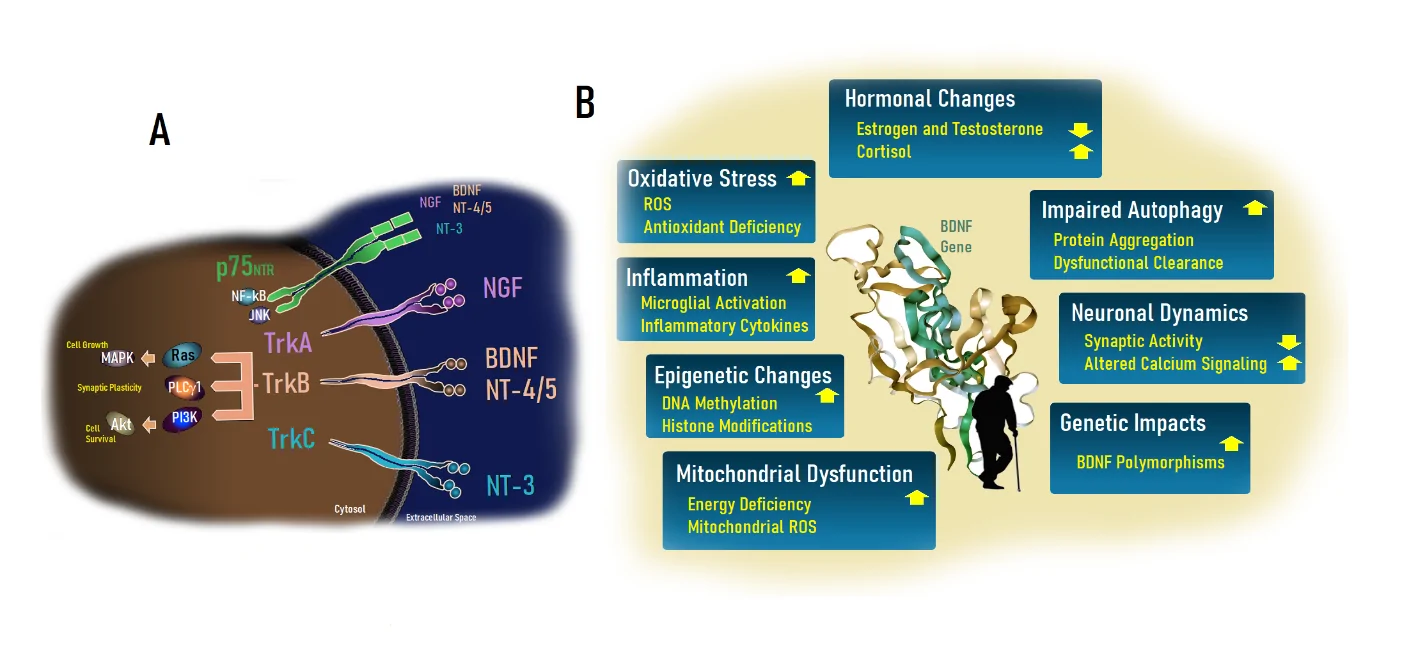
**BDNF signaling and the aging brain**. (**A**) *Neurotrophins, neurotrophin receptors and their downstream signaling pathways.* Neurotrophins are a family of proteins that play crucial roles in the development, maintenance, and survival of neurons in the nervous system. BDNF is one of neurotrophic proteins that supports the survival, growth, and differentiation of neurons in the central nervous system. It is crucial for synaptic plasticity, which underlies learning and memory processes. Note that mature BDNF and NT-4/5 bind to the TrkB receptor. Also, all members of the neurotrophin family are capable of signaling through a low-affinity receptor, referred to as the p75 (p75_NTR_) receptor, which is structurally distinct from the Trk receptors. NGF, *nerve growth factor*; NT, *neurotrophin*; BDNF, *brain-derived neurotrophic factor*; TrK, *tropomyosin receptor kinase*; PLCγ1, *phospholipase* Cγ1; PI3K, *Phosphatidylinositol-3-kinase*; MAPK, *mitogen-activated protein kinase*; NF-κB, *nuclear factor-κB*; JNK, *Jun kinase*. (**B**) *Principal mechanisms of BDNF decline in the aging brain*. The decline of BDNF in the aging brain is multifactorial. BDNF levels tend to decrease in the aging brain, contributing to increased susceptibility to neurodegenerative diseases, cognitive decline, reduced overall resilience and lower adaptation to life adversities.

## Neurotrophins, guardians of nervous system function

Neurotrophic proteins are among the most structurally complex and functionally sophisticated molecules critical for the proper functioning of the nervous system. They are involved in a wide range of processes, including neuronal growth and differentiation, synaptic plasticity, mood regulation, and the brain's response to injury and disease [66-69]. The most well-known neurotrophins include nerve growth factor (NGF), BDNF, neurotrophin-3 (NT-3), and neurotrophin-4/5 (NT-4/5). Each of these neurotrophins interacts with specific receptors on the surface of neurons to exert their supportive effects. For instance, NGF primarily binds to the tropomyosin receptor kinase A (TrkA) receptor, while mature BDNF and NT-4/5 bind to the tropomyosin receptor kinase B (TrkB) receptor. NT-3, on the other hand, specifically recognizes TrkC receptors [70]. It is also important to note that all members of the neurotrophin family are capable of signaling through a low-affinity receptor, referred to as the p75 (p75_NTR_) receptor, which is structurally distinct from the Trk receptors [see [71] for further discussion] (Fig. 2A). Although p75_NTR_ exhibits lower affinity compared to the Trk receptors, it is a multifunctional protein that intimately interacts with neurotrophins and other ligands to regulate various aspects of neuronal development, survival, and function, especially in aging.

When comparing BDNF with other neurotrophins such as NGF, NT-3, and NT-4/5, several key differences and similarities emerge, especially concerning their effects on neural resilience and aging. For instance, NGF levels, although decreasing with age, particularly affect basal forebrain cholinergic neurons, which are recognized as an NGF-sensitive cell population. Hence, NGF loss may lead to cognitive impairments, especially in Alzheimer's disease (AD) [[72]; see also [73] for more discussion]. Specifically, the NGF signaling system, in which TrkA or NGF-specific receptors are expressed, appears to be dysfunctional during the prodromal stages of AD, contributing to the selective degeneration of nucleus basalis cholinergic cortical projection neurons in AD [74]. Unlike BDNF and NGF, the role of NT-3 and its receptor TrkC in aging is less understood, although its expression seems to be higher in the CNS during the intrauterine period than in the adult brain [75], thus promoting survival of several neuronal populations in early development. NT-3 has also been shown to be involved in the survival of motor peripheral nerves [76] and proprioceptive neurons [77, 78]. NT-4/5, however, has a more limited distribution compared to BDNF, even though it still plays a role in synaptic plasticity and neuron survival. Like NT-3, the effects of NT-4/5 on aging are also not as well studied as those of BDNF or NGF. However, its decline with age could potentially contribute to memory deficits [79] and specific forms of neural degeneration, particularly in the sensory systems [80]. It appears that NT-4/5 expression is more region-dependent and exhibits differential effects depending on the timing and location of its co-expression with BDNF [81]. Moreover, it is noteworthy that in the realm of neurotrophins, BDNF emerges as particularly paramount due to its diverse and impactful function, especially in the context of experience-dependent aging and associated changes in the brain.

## BDNF, tiny but mighty

BDNF [82], a small secreted protein, is the brain's fertilizer, nurturing growth, adaptation and resilience [83]. The factual rationale that supports such impressions is that neurotrophins such as BDNF control numerous cellular functions, including proliferation, differentiation, migration, and survival in the brain. Interestingly, BDNF is synthesized and released constitutively [84] and in an activity-dependent manner suggesting that endogenous extracellular BDNF levels are extremely low [82] and its release and regulation is highly dependent on exogenous stimulus patterns [85]. The synthesis of BDNF within the CNS occurs in the form of its precursor, proBDNF, which exhibits the capacity for storage within dendrites or axons. The precursor proBDNF subsequently undergoes cleavage processes, both intra- and extracellularly, to generate the mature BDNF protein [86]. Notably, proBDNF and mature BDNF represent two different functional states of the BDNF protein with distinct roles [87-89] in regulating neuronal structure and function. For example, proBDNF exhibits a preference for binding to the p75_NTR_ receptor, which can be associated with facilitating long-term depression (LTD) [90] and apoptosis [91], whereas mature BDNF binds selectively to TrkB receptors which promote cell survival, facilitate long-term potentiation (LTP) and increase spine complexity [86]. In the aging brain, there tends to be an imbalance between proBDNF and mature BDNF levels, with an increase in the ratio of proBDNF to mature BDNF, a pathogenic process which is thought to contribute to aging-related cognitive decline and neuronal dysfunction [92].

At the genetic level, the BDNF gene in humans is localized on chromosome 11p14.1 [93]. A single-nucleotide polymorphism in the proregion of BDNF, known as the *Val*66*Met* polymorphism, involves a substitution of methionine (*Met*) for valine (*Val*) at position 66 in the BDNF protein. This change can affect the intracellular processing and the activity-dependent secretion of BDNF [94]. Specifically, the presence of the *Met* allele is associated with impaired trafficking of BDNF-containing vesicles to the synaptic site, leading to reduced release of BDNF during neuronal activity. Individuals with the *Met* allele generally exhibit reduced BDNF secretion in response to neuronal activity, which affects synaptic plasticity and cognitive function [95, 96]. Consequently, lower levels of BDNF in *Met* allele carriers may be associated with decreased resilience to age-related cognitive decline, increased susceptibility to stress, diminished psychological resilience, and a higher risk of developing neurodegenerative conditions such as Alzheimer’s disease in the elderly. Hence, the *Val*66*Met* polymorphism affects the production and release of BDNF, and may represent a key factor in understanding individual differences in aging trajectories and susceptibility to age-related cognitive and mood disorders ([97]; see also [98] for further discussion).

## BDNF, resilience, and challenges of brain aging

Brain aging is accompanied by several challenges to resilience. As explained earlier, resilience represents a dynamic process that involves the existing neurobiological potentials for optimizing adaptive responses to aversive experiences over the life course. Both development and lifetime adaptation to a dynamically changing environment are directly linked to BDNF activity in the brain. BDNF levels in the brain fluctuate throughout postnatal development. However, the regulation of BDNF in the aging brain appears to vary depending on the region and species [99], with some regions showing upregulation while others displaying downregulation. Since the brain serves as the central hub for perception and response to adversities, it is highly susceptible to the effects of allostatic load, with a similar level of vulnerability observed also in other biological systems (or organs) in response to stressors [100]. The region-dependent alterations in BDNF and the vulnerability of brain aging to the cumulative wear and tear (i.e., allostatic load/overload) can be traced through the neuroendocrineimmune, metabolic, and behavioural phenotypes in the elderly. Here, we briefly survey the most influential, deleterious neurobiological events in the brain that proactively play as drivers of brain aging. In this section, we also discuss how changes in BDNF deal with the destructive neuronal dynamics to support neuronal repertoires in favor of an optimal function. Three major domains of experimental observations related to brain aging and reduced resilience in the elderly, i.e. neuronal events and dynamics, inflammatory processes and neuroinflammation, and blood-brain barrier dysfunction and morphological changes, will be discussed. Indeed, aging is a complex biological process that should be examined comprehensively. Yet, delving into individual cellular and/or molecular events that accelerate brain aging or affect overall resilience in older individuals is crucial for gaining insight into aging and initiating innovative anti-aging strategies.

### 1. Neuronal events and dynamics

Brain aging is characterized by shifts in the functional properties and dynamics of neurons, which may have maladaptive consequences. Typically, the brain encounters neuronal dysfunctions such as neurodegeneration, decreased neuroplasticity, and the accumulated impacts of oxidative stress as it ages, which coincide with disruptive inter-neuronal dynamics within the connectome. These changes closely contribute to declines in cognitive abilities, impaired mobility, and unfavorable mood alterations [5, 101]. Importantly, age-related alterations in neuronal function can diminish resilience [102, 103]. While not within the immediate focus of this review, it still remains essential to address these cellular changes, particularly when examining the beneficial impact of BDNF on optimizing resilience during aging. Numerous abnormal processes (Fig. 2B) lead to neuronal dysfunction in aging, which can potentially be modulated by BDNF and BDNF-mediated regulatory mechanisms:

### 1.1 Neurodegeneration (gradual loss of neurons)

Neurodegeneration is one of the biological hallmarks of brain aging. Two observations regarding aging, neurodegeneration and resilience are of significance. One, neurodegeneration occurs over time; that is, some degree of neuronal loss and decline in brain function is considered a normal part of aging [104]. Second, neurons are selectively vulnerable in different neurodegenerative diseases [105]. However, excessive or accelerated neurodegeneration can lead to region-dependent neuropathology, which is typically associated with cognitive decline and/or motor dysfunction, and ultimately neurodegenerative diseases. Notably, neurodegeneration in the aging brain can be accelerated by several mechanisms.

The aging brain experiences compromised protein clearance mechanisms [106]. Normally, the brain employs mechanisms for the degradation of cellular proteins to remove damaged or misfolded proteins. These mechanisms include the ubiquitin-proteasome system (UPS) [107, 108] and autophagy-lysosomal pathways (ALP) [109, 110]. Aberrations in either mechanism may result in disruptions to protein quality control and homeostasis, thereby contributing to neurodegenerative conditions [111]. Notably, both protein degradation systems involve the BDNF/TrkB neurotrophic signaling in various functional aspects within the aging brain, particularly through neuroplasticity-related processes (see section *1.2*). In many neurodegenerative diseases, abnormal amounts or misfolded proteins accumulate within or between neurons, predominantly due to the loss of proteostasis (i.e., protein homeostasis) [104, 112]. The accumulation of toxic proteins may include existence of extracellular amyloid-β (Aβ) plaques and intraneuronal neurofibrillary tau tangles in AD [113-115], aggregation of alpha-synuclein (α-syn) in nigrostriatal neurons in Parkinson's disease (PD) [116, 117], and accumulation of huntingtin protein in Huntington's disease (HD) [118, 119]. These protein aggregates may disrupt normal cellular function and lead to neuronal damage at different levels of neuropathological events such as DNA damage, and mitochondrial and lysosomal dysfunction. It is important to note that aging-related protein abnormalities, especially in the phagolysosomal system within the aging brain, result in blurred distinctions between aging and neurodegenerative disorders. Consequently, elderly individuals may have pathological abnormalities in the brain that do not necessarily correlate with their cognitive disabilities or go undiagnosed [104].

AD patients typically suffer from reduced BDNF [120]. Notably, neurotrophin replacement therapy to the brain is challenging. Drug delivery to the CNS through peripheral administration is associated with complications in part because only minimal quantities of BDNF can cross the blood-brain barrier (BBB) due to its charge and molecular dimensions [121]. Using an alternative procedure known as adeno-associated virus (AAV) injection carrying the BDNF gene [122], several studies paved the way for AAV-based gene therapies that are associated with minimal complications and maximal efficacy [123, 124]. An alternative procedure has been recently suggested for targeted delivery of the BDNF gene to the brain using liposome nanoparticles to increase BDNF protein levels and reverse AD pathophysiology [125]. Of note, BDNF gene delivery involves several delivery vectors with different advantages and disadvantages. Subclinical manipulations aimed at infusing AAV-BDNF gene into the intralateral ventricle of the brain resulted in the restoration of the BDNF level, along with alleviated synaptic degeneration and attenuated behavioural deficits [122]. However, the gene delivery of BDNF did not affect tau hyperphosphorylation levels in AD. It appears that BDNF itself does not directly degrade or remove toxic proteins, such as the tau protein, from the brain [126]. Yet, the efficacy of BDNF manipulations in AD suggests that BDNF-involved mechanisms such as the BDNF/TrkB signaling pathway can modulate Aβ-induced neurotoxicity in AD and other neurodegenerative conditions with tauopathy [see [127] for more discussion].

Furthermore, α-syn protein overexpression is associated with the downregulation of BDNF mRNA and protein in the pars compacta (SNpc) of the substantia nigra in patients with PD, and lower levels of BDNF can increase vulnerability to degeneration in nigral neurons [128, 129]. On the other hand, selective α-syn overexpression may negatively impact BDNF signaling and/or reduce BDNF gene and protein expression [130]. Using an α-syn-HDO that specifically targets α-syn abnormal overexpression, it has been recently shown that α-syn silencing protects dopaminergic neurons from degeneration via activation of BDNF transcription [129]. α-syn silencing may also upregulate BDNF mRNA expression [131], increase TrkB protein levels, and BDNF/TrkB neurotrophic signaling along with restoring motor function [85, 132]. If the pathological accumulation of α-syn protein leads to a reduction in BDNF levels, it suggests that BDNF may potentially influence disease progression in PD by regulating α-syn levels.

Criticism has recently been raised against neuroprotective approaches to PD due to their limitations in addressing the multifaceted nature of this condition, constraining it within the framework of a single pathogenic disease entity [133]. Therefore, it is recommended that interventions targeting PD pathogenesis be tailored to accommodate potential efficacy according to the distinct biological processes evident in various subgroups of PD patients. However, BDNF-centered interventions may still offer opportunities for exemption from these limitations, owing to BDNF's wide-ranging biological effects. Indeed, BDNF may indirectly influence α-syn clearance mechanisms in PD. BDNF may slow apoptotic processes in dopaminergic neurons in SNpc and enhance protein degradation pathways, including autophagy, which plays a role in clearing misfolded proteins [134, 135]. Although not specific to PD, BDNF-involved neuroprotective interventions provide insights into potential mechanisms by which BDNF induction may confer neuroprotection and modulate protein aggregation pathways.

Brain aging is also associated with an increase in oxidative stress, which can damage neurons and impair their function over time [136]. The free radical theory of aging [137] suggests that aging and associated diseases are caused by damage accumulated over time from free radicals or reactive oxygen species (ROS) in the body. In fact, ROS are the natural byproducts of cellular metabolism that can impair various neuronal components such as DNA, proteins, and lipids if not adequately neutralized by antioxidants. This damage, known as oxidative stress, can lead to impaired cellular function and eventually contribute to aging and age-related diseases like neurodegenerative disorders, which usually result from the disruption of oxidant-antioxidant balance in neurons or an excess of ROS accompanied by a compromised intrinsic antioxidant defense [138]. Accordingly, the decline in antioxidant defenses drives the aging brain to increased oxidative stress and damage to neuronal architecture. BDNF, however, may counteract this process [6, 55]. For instance, BDNF/TrkB signaling attenuates oxidative stress by enhancing mitochondrial function and biogenesis through the upregulation of thioredoxin and peroxiredoxins [56], which are involved in maintaining cellular redox balance, and by activating peroxisome proliferator-activated receptor gamma coactivator 1-alpha (PGC-1α) [139]. This reduction in oxidative stress is critical for maintaining cellular homeostasis and protecting against age-related neurodegeneration. More importantly, BDNF shares overlapping roles with sirtuins (SIRT1-SIRT7) in regulating cellular processes in brain aging. SIRTs are histone deacetylases (HDACs) whose activity depends on the cellular metabolic status and regulate energy metabolism and mitochondrial function [140]. SIRT1 activation may upregulate BDNF expression, potentially mitigating aging-related cognitive decline [[141, 142], See [143] for further discussion]. In humans, the overactive allele of SIRT6, which is known to regulate both longevity and progeria through the control of oxidative homeostasis [144, 145], is associated with longer life [146]. SIRT6 also plays a critical role in DNA repair, telomerase function, genomic stability, and cellular senescence [147], all of which involve BDNF function. Interestingly, caloric restriction that stabilizes mitochondrial function and reduces oxidative stress [148-150], has a potent antioxidant correlate: BDNF [151].

Dysregulation of mitochondrial function and dynamics in the aging brain [152] signifies diminished energy metabolism within the neurons. Mitochondrial dysfunction can impair energy production, induce lipid peroxidation and heighten oxidative stress [137, 153, 154], thus exacerbating neuronal damage and precipitating cognitive decline in aged individuals. BDNF modulates mitochondrial physiology by enhancing mitochondrial biogenesis (i.e., the generation of new mitochondria) [155]. The neuroprotective effects of BDNF on mitochondrial function was also shown to be associated with increased resistance of neurons to oxidative stress [156]. BDNF is a key player to regulate mitochondrial dynamics by influencing the expression and activity of proteins involved in fusion (e.g., mitofusins) and fission (e.g., dynamin-related protein 1). Of note, such functional dynamics are essential for maintaining a healthy mitochondrial network and optimal cellular function. BDNF can influence the transport of mitochondria within neurons along axons and dendrites (see *1.2*). Therefore, dysregulated communication between BDNF and mitochondria may severely disrupt cellular energy metabolism and contribute to the pathogenesis of neurological disorders, especially neurodegenerative diseases.

The process of brain aging is also associated with complex changes in hormonal and neurotransmitter systems. Alterations in the concentrations and functioning of various neurotransmitters, e.g., dopamine [[157], serotonin [158], and acetylcholine [159], see [160] for more discussion] impact mood, cognition, and behaviour in the elderly. However, excessive activation of excitatory neurotransmitters, primarily glutamate [161], through excitotoxicity can specifically lead to neurodegeneration and neuronal death by increasing intracellular calcium concentrations in neurons [84, 162]. Aging-related changes in neurotransmitter systems and receptor sensitivity may increase susceptibility to excitotoxicity. It appears that regulation of glutamate homeostasis (release, uptake, and metabolism) to some extent depends on the BDNF/TrkB neurotrophic signaling in the brain. This signaling pathway regulates the expression of glutamate transporters and receptors, ensuring proper clearance of glutamate from the synaptic cleft and preventing its accumulation to toxic levels [163]. The multifaceted nature of neurodegeneration in aging brains underscores the intricate interplay between various biological processes and pathological mechanisms. While challenges remain in tailoring interventions to accommodate the heterogeneous nature of neurodegenerative conditions, the wide-ranging biological effects of BDNF provide hope for therapeutic strategies that address the complex underlying mechanisms of aging-related neurodegeneration and reduced resilience in the elderly.

### 1.2 Decreased Neuroplasticity

Neuroplasticity enables the brain to learn, adapt, and change (e.g., recover) in response to experiences and environmental influences [42, 164]. As people age, neuroplasticity tends to decline, making it more difficult for the brain to form new connections and reorganize neural pathways. Like neurodegeneration, diminished neuroplasticity in older adults may compromise cognitive functions such as learning, memory, and other cognitive processes linked with cognitive aging [165]. The mechanisms through which the BDNF/TrkB neurotrophic signaling preserve optimal neuroplastic processes during aging seem to involve several key neurobiological dynamics. However, the most important domains of reported observations include synaptic plasticity (synaptic transmission, and changes in spine number, size, and shape), neurogenesis, and neuronal survival and anti-apoptotic mechanisms.

*Synaptic plasticity.* In addition to its neuroprotective function, BDNF serves as a biomarker for neuronal plasticity [84]. It promotes synaptic plasticity by enhancing the formation and strengthening of synapses [166], thereby maintaining cognitive function and memory as the brain ages. Conversely, reduced BDNF signaling was shown to directly contribute to low synaptic function through altered expression of markers for inhibitory and excitatory neurons in human brain aging and synaptic function-related genes [167]. The BDNF signaling in regulating synaptic transmission and dendritic integrity holds significance due to two distinct findings. Firstly, it manifests in both presynaptic and postsynaptic neuronal compartments. Secondly, BDNF mRNA can be transported in neuronal dendrites in an activity-dependent manner [168, 169]. Accordingly, age-dependent decrease in BDNF signaling may cause synaptic alterations, the most consistent plasticity-related deficits associated with poor cognitive resilience [e.g., cognitive flexibility and perspective-taking [26]] and other forms of cognitive performance such as learning and memory in the elderly. For example, BDNF-LTP in different cerebral regions is impaired in the aged brain, and can be restored by endogenous BDNF induction through pharmacological approaches [168, 170] and physical exercise regimens [171].

LTP and LTD, two models of synaptic plasticity, represent the ability of synapses to strengthen or weaken over time in response to activity. LTP involves the strengthening of synaptic connections between neurons when two neurons are stimulated simultaneously, leading to enhanced interneuron communication. LTD, on the other hand, is the long-lasting decrease in synaptic strength, and involves the weakening of synaptic connections between neurons [16, 172]. In a neurofunctional perspective, LTP is typically induced by high-frequency stimulation of the presynaptic neuron, causing a significant influx of calcium ions into the postsynaptic neuron. Consequently, the influx of calcium triggers various molecular mechanisms, including the activation of calcium-calmodulin-dependent protein kinase II (CaMKII), the insertion of additional the α-amino-3-hydroxy-5-methyl-4-isoxazolepropionic acid (AMPA) receptors into the postsynaptic membrane [173, 174], and an increase in the transcription of postsynaptic BDNF mRNA [175] as a retrograde messenger [173]. However, LTD results in a decrease in the number or efficacy of postsynaptic receptors, such as the removal of AMPA receptors from the postsynaptic membrane, thus refining neural circuits and eliminating unnecessary connections. Both processes can last for extended periods of time and have been shown to optimize synaptic transmission and cognitive function through increased synaptic plasticity. However, biological age dramatically determines LTP and LTD functions in the brain through dysregulating p75_NTR_ [176]. It appears that the aging neurons display an increased tendency to induce LTD and reverse LTP, driving the decrease of synaptic transmissions [177]. Nevertheless, when locally microinfused, BDNF can trigger long-lasting synaptic strengthening in a region-dependent manner within the hippocampus, and selective induction of the dendritic mRNA species activity-regulated cytoskeleton-associated protein (Arc) [178].

Notably, BDNF also facilitates the transport of mitochondria within neurons along axons and dendrites. This transport is crucial for providing energy to distant neuronal compartments and supporting synaptic transmission and plasticity. By regulating mitochondrial motility [179], BDNF ensures adequate energy supply to active synapses and promotes neuronal communication and connectivity. Due to its critical neurotrophic roles in synaptic integrity and function, BDNF can be an essential component of the cellular mechanism supporting memory formation and maintenance by promoting synaptic consolidation, thus enhancing cognitive resilience in aging.

### Neurogenesis

Neurogenesis, the process by which new neurons are generated in the brain [180], tends to decline in certain brain regions during biological aging [181, 182]. Our understanding regarding the mechanisms by which neurogenesis facilitates cognitive functions and resilience in healthy adults is still in its nascent stages. More importantly, how and to what extent decreased neurogenesis contributes to cognitive decline in aging appears to be even more elusive. BDNF stimulates neurogenesis [183], particularly in regions that are intimately involved in learning and memory such as the hippocampus [184], offsetting aging-related neuronal loss and supporting cognitive resilience. Subclinical observations suggest that the subventricular zone (SVZ) of the lateral ventricles, and the subgranular zone (SGZ) in the hippocampal (HPC) dentate gyrus are the main cerebral regions for adult neurogenesis. These areas are known to provide neural stem/progenitor cells (NSPCs) that generate neuroblasts undergoing neuronal differentiation. Neurogenesis within the SVZ also supplies fresh interneurons to the olfactory bulb [185, 186]. It appears that age is a co-factor that plays a key role in smaller HPC volumes (an indicator of diminished neurogenesis), reduced levels of serum BDNF, and poor memory performance in older adults [187]. Increased HPC volume, on the other hand, is associated with greater serum levels of BDNF and improved spatial memory in the elderly [188]. BDNF has been also shown to stimulate the proliferation of NSPCs in the brain, precursor cells capable of differentiating into various types of neurons. By increasing the pool of NSPCs, BDNF creates more opportunities for neurogenesis to occur. Within the revolutionary concept of induced glia-to-neuron conversion [189, 190], originally proposed independently of neurotrophic signaling in the brain, proliferative neuroblasts generated by reprogrammed resident astrocytes have been shown later to develop into mature neurons and functionally integrate into the local neural network when supplied with BDNF [191]. Noteworthy, the significance of neurotrophins in such processes lies in their most characteristic functional aspect, which can be shared with neurogenesis as the fundamental mechanism: both BDNF neurotrophic signaling and neurogenesis represent activity-dependent cellular/molecular dynamics within the brain, highlighting their common vulnerability to activity-dependent changes or exogenous stimulations (e.g., exercise, social support, pharmaceutical intervention, dietary and environmental enrichment) in enhancing neurogenesis and cognitive resilience in older adults ([192, 193], also see [194] for further discussion) (BOX 3).

### Neuronal survival and anti-apoptotic mechanisms

The integrity of the BDNF/TrkB pathway and its multifaceted role in enhancing resilience against age-related neuropathogenesis are particularly vital for neuronal viability [195] and anti-apoptotic mechanisms [196, 197] in the aging brain. These mechanisms are typically associated with the activation of downstream signaling cascades such as the phosphoinositide 3-kinase (PI3K)/Akt and mitogen-activated protein kinase (MAPK)/extracellular signal-regulated kinase (ERK) pathways [198, 199]. Upon BDNF binding to the TrkB receptor and the subsequent autophosphorylation of TrkB on specific tyrosine residues within its intracellular domain, PI3K, a critical downstream signaling molecule, is recruited to and activated by the phosphorylated TrkB receptor. The activation of PI3K leads to the production of phosphatidylinositol-3,4,5-trisphosphate (PIP3), a second messenger that recruits Akt (also known as protein kinase B, PKB) to the plasma membrane. Activated Akt plays a central role in promoting neuronal survival by phosphorylating, inactivating, and/or inhibiting several pro-apoptotic factors, such as the pro-apoptotic Bcl-2-associated death promoter (Bad) and glycogen synthase kinase-3β (GSK-3β), a kinase implicated in promoting apoptosis [200]. The MAPK/ERK pathways activated by BDNF/TrkB signaling also inhibit apoptotic signaling by, for instance, upregulating anti-apoptotic proteins of the Bcl-2 family [201] and downregulating pro-apoptotic factors such as Bax [202-204]. The MAPK/ERK pathway is initiated when TrkB activates Ras (Fig. 2A), a small GTPase, through the recruitment of the adaptor proteins Shc and Grb2, along with the guanine nucleotide exchange factor SOS. Activated Ras then triggers a kinase cascade involving the sequential activation of Raf, MEK (mitogen-activated protein kinase kinase), and ERK (the Ras/Raf/MEK/ERK signaling cascade) [205]. Activated ERK translocates to the nucleus [206], where it phosphorylates and activates transcription factors such as CREB (cAMP response element-binding protein). CREB activation not only leads to the upregulation of anti-apoptotic genes [207, 208] but also promotes the transcription of BDNF genes themselves [209, 210], enhancing neuronal resistance to apoptotic stimuli such as aging and creating a positive feedback loop that sustains neurotrophic support. More importantly, significant crosstalk appears to exist between the PI3K/Akt and MAPK/ERK pathways [211], which enhances the robustness of the survival signals initiated by BDNF/TrkB signaling. For instance, Akt can phosphorylate and inhibit components of the MAPK pathway to fine-tune the cellular response. Conversely, ERK can modulate the activity of certain Akt targets, creating a coordinated response that optimizes neuronal survival [212]. In the aging brain, the efficacy of these survival pathways is often diminished due to reduced BDNF levels and impaired TrkB receptor signaling, which compromises the ability of neurons to effectively engage these survival pathways. However, maintaining or restoring BDNF/TrkB signaling has been shown to counteract these age-related deficits [[199], see also [213] for further discussion], and preserve neuronal survival and resilience in the aging brain.

**Figure 3.**
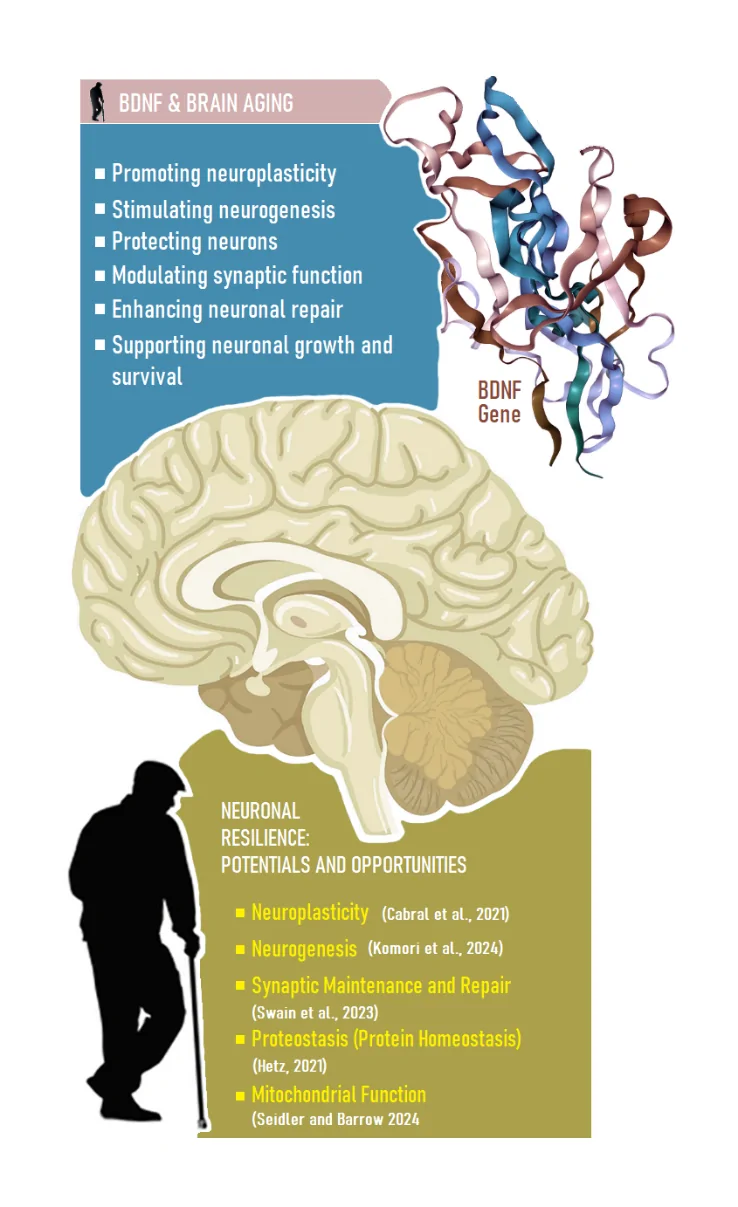
**BDNF and neuronal resilience in the elderly**. BDNF plays a crucial role in supporting neuronal resilience, especially in the aging brain. Neuronal resilience refers to the brain's ability to resist, adapt to, and recover from various stressors, including aging-related changes. As the brain ages, it undergoes numerous structural and functional modifications. However, certain mechanisms and factors contribute to maintaining neuronal resilience, thus helping preserve cognitive functions and overall brain health.

**BOX 3: Overview:** *Neurotrophic Hypothesis, BDNF, and Brain Aging* if BDNF can activate neuronal survival mechanisms, it may also possess the potential to enhance neuronal resilience within the aging brain (Fig. 3). Previous hypotheses suggest that deficiencies in neurotrophic support, including impaired BDNF function, could be a significant factor contributing to depression [271-273], leading to pathological deterioration and behavioural decline. Conversely, augmenting neurotrophic support could potentially alleviate these symptoms [273]. When considering neuronal resilience within the aging brain, the neurotrophic hypothesis can be applied through several mechanisms that directly involve BDNF action in the brain: (*i*) *Promoting neuroplasticity.* Aging brains experience a decline in neuroplasticity, which can contribute to cognitive decline and increased vulnerability to neurodegenerative diseases. Interventions aimed at enhancing neurotrophic factor signaling may help maintain or restore neuroplasticity, thus promoting resilience to aging-related cognitive decline [228, 274, 275]. (*ii*) *Stimulating neurogenesis.* Neurogenesis declines with age, mainly due to a decrease in the quantity of neural stem/progenitor cells (NSPCs) and transit amplifying progenitor cells (TAPs) [276]. However, neurogenesis persists to some extent in specific brain regions, such as the hippocampus. BDNF has been shown to create a conducive environment for the generation of new neurons and their integration into existing neuronal circuits [234, 277]. Strategies that enhance neurotrophic factor signaling could potentially promote neurogenesis in the aging brain, contributing to neuronal resilience. (*iii*) *Protecting neurons against degeneration (cellular resilience).* Neurotrophic factors have neuroprotective effects, shielding neurons from various insults such as oxidative stress, inflammation, and protein aggregation, which are common features of aging and neurodegenerative diseases [278, 279]. Neurotrophic factors may also promote the clearance of toxic protein aggregates (protein degradation), such as beta-amyloid in Alzheimer's disease or alpha-synuclein in Parkinson's disease, further contributing to neuronal protection [56]. Enhancing the availability or activity of neurotrophic factors may help mitigate aging-related neuronal damage and promote neuronal resilience. (*iv*) *Modulating synaptic function.* Neurotrophic factors such as BDNF and nerve growth factors (NGF) influence synaptic structure and function, including synapse formation, maintenance, and plasticity [156]. Aging-related changes in synaptic connectivity and function contribute to cognitive decline and vulnerability to neurodegenerative diseases. Interventions targeting neurotrophic factor signaling pathways may help preserve synaptic integrity and function, thus fostering neuronal resilience. (*v*) *Enhancing neuronal repair and recovery*. In response to injury or aging-related changes, BDNF/TrkB signaling can help facilitate brain repair and recovery by promoting synaptic remodeling, and functional reorganization [167, 280, 281]. BDNF may help recover impaired neurons in the aging brain through a close collaboration with M2 microglia [282]. Microglia are the resident immune cells of the central nervous system, and their activation state is generally associated with anti-inflammatory and tissue repair functions. M2 microglia, an alternative microglia phenotype, typically produce anti-inflammatory cytokines (IL-10 and IL-4) and neurotrophic mediators such as BDNF and promote tissue repair [223, 283]. M2 microglia are involved in processes such as synaptic pruning, neurogenesis, and the resolution of inflammation, contributing to repair of neural tissue damage. This ability to support brain plasticity is crucial for maintaining cognitive function and resilience in the face of aging-related challenges. (*vi*) *Neuronal growth and survival*. BDNF supports the growth and survival of neurons [284], particularly in areas important for learning and memory, such as the hippocampus [285]. In the aging brain, where neuronal connections may weaken or decline, BDNF may help stimulate the growth of new neurons and strengthens existing connections [286]. Given the importance of neurotrophic factors in maintaining neuronal health and function, therapeutic interventions aimed at enhancing neurotrophic factor signaling [284] represent a promising approach for promoting neuronal resilience in the aging brain. These strategies may include pharmacological agents, such as small molecule agonists or gene therapy approaches aimed at increasing endogenous neurotrophic factor production or enhancing receptor signaling, potentially contributing to the preservation of cognitive function and resilience in the aging brain.

### 2. Inflammatory processes and neuroinflammation

The hallmarks of aging have been recently updated to include chronic inflammation [8]. A concise biological interpretation of such concepts is that persistent low-grade inflammation, a sophisticated biological condition termed as inflamm-aging, increases with aging [50]. Inflamm-aging, which typically occurs in the absence of overt signs, and immunosenescence collectively underlie the genesis of most diseases and compromised resilience in the elderly [46]. Additionally, inflamm-aging constitutes a hallmark feature of the aging cerebral milieu. Notably, low-grade inflammatory reactions are commonly termed neuroinflammation when localized in the brain and spinal cord. Such responses in the aging brain primarily involve a cascade of detrimental biochemical reactions that may occur locally in glial populations, particularly in microglia (the brain-resident macrophages). These responses are characterized by the acquisition of an ameboid morphology, the imbalanced production of pro-inflammatory cytokines and chemokines, as well as the overproduction of ROS [214-217]. The overall role of microglia in neuroinflammation is beyond the scope of this discussion. However, it is important to briefly note that the extent to which neuroinflammation impacts neuronal dynamics (e.g. neurogenesis in AD) in the aging brain depends upon the duration and intensity of microglial activation in response to injury or disease. Chronic neuroinflammatory processes can contribute to neuronal dysfunction and abnormal neural signaling [218], suppressed neurogenesis [219] and neurodegeneration [220] in the aging brain. Accordingly, it is not surprising that neuroinflammation and its subsequent maladaptive functional impacts on brain aging have been implicated in the pathogenesis of many diseases in aged individuals. Nevertheless, findings obtained from diverse interventions, such as caloric restriction, putative neuroprotective interventions by BDNF, and exercise, aimed at reducing aging-related brain pathologies, challenge the prevailing concept of brain aging as a rigid process [6, 221, 222].

BDNF is a prompt synergist of microglial cells [[223], see [224] for more discussion]. Besides its well-known, potent neurotrophic effects, BDNF can modulate inflammatory processes in various tissues and organs, including the brain, even though its anti-inflammatory properties are not as extensively studied as its neurotrophic effects. Preclinical observations indicate that BDNF treatment reduces degrees of microglial activation in the presence of the brain injury along with reduced neuronal injury and death [225-227]. Even though the direct effect of BDNF administration on microglia has rarely been explored, recent findings show that the upregulation of BDNF signaling inhibits aging-related microglial activation via the TrkB-Erk-CREB pathway in aging mice [195]. Interestingly, suppressing BDNF/TrkB signaling during aging was associated with microglial activation, indicating a direct antimicroglial activation effect of BDNF in the aging brain [195].

In the aging brain, microglia have also been shown to play a key role in astrocyte reactivity via the secretion of pro-inflammatory cytokines and components of the complement system [5, 218]. BDNF, on the other hand, has been suggested to inhibit the activation of pro-inflammatory signaling pathways [228]. This includes pathways mediated by cytokines such as tumor necrosis factor-alpha (TNF-α) and interleukin-6 (IL-6), which are implicated in neuroinflammation. Moreover, BDNF can upregulate the expression of anti-inflammatory cytokines such as interleukin-10 (IL-10) [229-231] and promote the activity of anti-inflammatory enzymes such as heme oxygenase-1 (HO-1) [232]. More importantly, BDNF has been shown to modulate the activation state of microglia, thus promoting a phenotype associated with reduced inflammation and enhanced neuroprotection [228, 233, 234]. By regulating microglial activation, BDNF can help maintain a balanced immune response within the aging brain. The concepts of inflamm-aging and neuroinflammation underscore the genesis of numerous diseases and compromised resilience in the elderly. Indeed, understanding the complex interaction among neuroinflammation, microglial activation, and the modulatory effects of BDNF offers promising avenues for therapeutic interventions aimed at mitigating neurodegenerative processes, cognitive decline, and promoting brain health in older adults.

### 3. Blood-brain barrier (BBB) dysfunction and morphological changes

The cerebral vascular system, which forms the BBB, is a highly specialized network of blood vessels responsible for preserving brain homeostasis and supplying oxygen, nutrients (glucose, amino acids, large neutral amino acids, etc.), and other essential substances to brain tissue while removing waste products [235]. The system and its vulnerability to aging-related changes have been recently discussed extensively by others [138, 236]. Growing evidence indicates that capillary vascular defects including BBB dysfunction contribute to many forms of brain pathologies such as neurodegeneration in aging. As individuals age, there are several changes that occur in all four types of BBBs [236] with a profound impact on their functions as a regulatory and protective network. Though some of these changes in the morphology and functionality of the BBB are adaptive, they can also closely contribute to vasculature deficits, which in turn may compromise cognitive performance, emotional integrity, and resilience in the elderly [235]. Similar to other complexities observed in the aging brain, changes in the aging BBB have not reached a clear consensus due to a simple, but critical observation: even in the absence of overt clinical or pathological manifestations, the human aging brain typically exhibits certain degrees of pathology. Ongoing advances in both clinical (e.g., rigorous physical examinations) and paraclinical (e.g. brain imaging, biomarkers) diagnostics may offer more comprehensive solutions to resolve these complexities in favor of enhancing the brain health and improving resilience in the elderly.

Two major manifestations of capillary vascular defects that may actively involve BDNF/TrkB signaling in the aging brain include permeability of BBB and metabolic changes. *Permeability of BBB*. The debate on whether BBB disruption occurs in the aging brain has persisted for decades. Nevertheless, aging-related changes in BBB have been repeatedly suggested to result in increased permeability (BBB leakage) [237-239], a process through which substances that would normally be restricted by the barrier may be able to pass through more easily. Consequently, harmful molecules such as toxins or pathogens as well as unregulated blood contents may gain access to the brain, potentially contributing to neurodegenerative dynamics or cognitive decline. Substantial evidence suggests that BDNF can support and maintain the integrity of BBB [240] by enhancing the expression of endothelial tight junction proteins (TJPs) that restrict paracellular permeability [241]. Disruption of the BBB is also associated with increased infiltration of inflammatory mediators and immune cells into the brain, contributing to neuroinflammation [242, 243]. This can create a feedback loop where inflammation further damages the barrier and causes more inflammation and barrier dysfunction. Accordingly, inflammatory processes in the aging brain affect the integrity of BBB, leading to increased permeability and dysfunction. However, BDNF can limit the influx of pro-inflammatory cytokines or circulating T-cells into the brain through maintaining the TJs stability and the regulation of neuroinflammation [240]. *Metabolic changes.* In addition to the BBB hyperpermeability induced by junction structural changes (e.g., TJPs instability), the aging brain experiences metabolic changes such as reduced expression of glucose transporter 1(GLUT-1) [244] and mitochondrial damage [235]; both defects have been reported to participate in brain pathogenesis and neurodegeneration during aging [154, 245], thus involving BDNF-dependent trophic processes.

GLUT-1 ensures that neurons receive an adequate supply of glucose to meet their energy demands. Hence, subtle deficits in GLUT-1 function in the brain may contribute to neurodegeneration [245]. BDNF has been shown to modulate the expression and activity of GLUT-1 at the BBB. Studies suggest that BDNF can upregulate GLUT-1 expression and enhance glucose transport and metabolism across the BBB [125, 246]. Conversely, reduced BDNF levels or impaired BDNF signaling may lead to decreased GLUT-1 expression and glucose transport, compromising neuronal energy metabolism and increasing susceptibility to age-related cognitive decline and neurodegenerative diseases [247]. More importantly, the functional symbiosis of BDNF and GLUT-l has been recently shown by Tang and others [248] where GLUT-1 depletion not only triggers a severe neuroinflammatory response in the brain, but also reduces levels of BDNF and causes overt disease. Also, since glucose uptake in microglia is facilitated predominately by GLUT-1, particularly under inflammatory conditions [249], and BDNF-microglia interplay is crucial to reduce neuroinflammation [223], the BDNF/TrkB signaling is the footprint of trophic dynamics in the presence of capillary vascular defects in the aging BBB. It appears that targeting the *BDNF-GLUT-1-Microglia* signaling pathway could be a constructive approach to control neuroinflammation and improve cognitive resilience during aging.

Alterations in mitochondrial function and dynamics also impair BBB integrity in the aging brain [250, 251]. BDNF has been shown to enhance mitochondrial function by increasing peroxisome proliferator-activated receptor-γ coactivator-1α (PGC-1α)-dependent mitochondrial biogenesis [139], improving ATP production, and reducing oxidative stress [155, 156]. This interaction is essential for maintaining cellular energy homeostasis and protecting neurons from aging-related damage. BDNF signaling pathways intersect with mitochondrial dynamics [252], including fission (mitochondrial division), fusion (mitochondrial interconnection), and mitophagy (recycling dysfunctional mitochondria) within the cerebral vasculature. An impaired balance between mitochondrial fission and fusion is one of the most characteristic mitochondrial defects in AD [253]. Additionally, all three mitochondrial dynamics - fusion, fission, and mitophagy- diminish with age, leading to a decline in mitochondrial quality in the aging BBB. However, BDNF promotes mitochondrial fusion and inhibits fission, leading to the formation of elongated and interconnected mitochondria, which are associated with enhanced bioenergetic capacity and resistance to stress [250, 254, 255]. Additionally, BDNF-mediated activation of mitophagy helps remove damaged mitochondria, thereby preserving overall mitochondrial function [139]. Furthermore, the BDNF-dependent trophic process to improve mitochondrial function can be facilitated through its well-known central anti-inflammatory effects. Since cerebrovascular inflammation disrupts BBB function in the aging brain [256] and vascular aging may impair neuronal health by reducing BBB integrity [250], BDNF is expected to play a critical role in mitigating these effects, potentially through its wide-spectrum effects on cellular energetics and metabolic homeostasis [252].

It is noteworthy that while substantial evidence supports a link between BDNF and specific aging outcomes, the field is also marked by several inconsistencies. Evidence positively linking BDNF to aging may include its role in enhancing cognitive performance by maintaining neuronal integrity, improving physical health, and protecting against emotional decline in the elderly. Inconsistencies in the evidence, however, are due to varying methodologies and differing study populations [257, 258]. For instance, although BDNF can be measured in serum, plasma, whole blood or cerebrospinal fluid (CSF), these methods often yield different results [259, 260]. This may lead to confusion about which biomarker is most reliable. Even within the same clinical population with different profiles of AD, serum BDNF could not predict disease group membership despite adequate power [261]. It is also notable that differences in study design, sample size, or population heterogeneity may further confound the reliability of results linking BDNF to aging outcomes. Importantly, lifestyle factors (e.g., exercise, diet) and genetics (e.g., BDNF Val66Met polymorphism) which can modulate the relationship between BDNF and aging may lead to variability in BDNF levels and complicate the interpretation of findings in older individuals. Furthermore, longitudinal studies tend to present an alternative picture of the associations between BDNF and aging outcomes compared to cross-sectional studies. Thus, the temporal dynamics of BDNF may also be crucial for understanding its role in aging [262-264]. It appears that these inconsistencies in the BDNF contribution in aging outcomes and resilience can be adequately addressed through standardized measurement techniques, larger and more diverse cohorts, as well as a better understanding of how BDNF interacts with other biological factors, particularly other neurotrophins and environmental contributors in the aging process.

## Concluding remarks

BDNF is the gardener of brain dynamics; it represents a critical component in the complex landscape of resilience and offers profound insights into the mechanisms underlying our capacity to withstand and overcome adversity. As a neurotrophin, BDNF exerts wide-ranging effects on brain architecture by impacting neuroplasticity, stress regulation, and emotional well-being. Why does BDNF emerge as a pivotal factor in fostering resilience in the aging trajectory? BDNF supports neuronal function and dynamics (survival of neurons, neuroplasticity, and neuroprotection) which are essential for maintaining cognitive function and resilience against aging-related cognitive and motor decline. Numerous findings also indicate that BDNF is involved in the regulation of the stress response system, including the HPA axis. BDNF helps to modulate stress hormone levels and attenuate the destructive effects of chronic stress, promoting resilience to stress-related disorders in aging. Moreover, aging involves a potent neuroinflammation dynamic in the brain which is directly accompanied by a heightened risk of neurodegenerative diseases. By reducing inflammation in the brain, BDNF contributes to resilience against cognitive decline and neurodegeneration associated with aging. Also, vascular pathology is a common feature of a range of neurodegenerative diseases that contribute to reduced resilience in aging. BDNF has been shown to promote vascular health by enhancing endothelial function and promoting angiogenesis. By improving blood flow and nutrient delivery to the brain, BDNF can support brain health and resilience against aging-related vasculature and metabolic challenges. Finally, BDNF plays a role in regulating mood and emotional resilience. Low levels of BDNF have been associated with mood disorders such as depression, while increased BDNF levels have been linked to improved mood and resilience to stress and negative emotions in aging adults [63, 65, 84, 166, 223].

The role of BDNF in brain aging is crucial, as it can significantly contribute to the development of enhanced therapeutic strategies and interventions. By understanding the mechanisms through which BDNF influences aging such as neuroprotection, mood regulation, neurogenesis, and cognitive function researchers can devise more effective approaches to maintain brain health, improve cognitive and emotional resilience, and potentially extend the healthy lifespan. If lower levels of BDNF are associated with smaller hippocampi and poor memory function in older adults [187], targeted interventions and therapies aimed at increasing BDNF levels can potentially counteract these age-related changes. However, though provocative, therapeutic approaches using BDNF-induced improvements for neurodegenerative diseases in late adulthood have encountered many challenges in recent years. For example, supplementing with exogenous BDNF presents several complications due to its molecular structure, which affects its ability to cross the blood-brain barrier [[265], see also [266] for a counterargument], as well as the pharmacokinetics and volume fluctuations of BDNF once it reaches the brain. These issues can lead to imbalanced BDNF/TrkB signaling and potential upregulation or downregulation of BDNF/TrkB-related pathways. Nevertheless, the use of low-dose BDNF sequentially kinetic activated (SKA), as an alternative delivery method, has recently been shown to counteract some mechanisms underlying the degeneration and aging of nervous tissue [6]. Also, given the regional variation in BDNF molecule concentration in the brain [188], which can contribute to region-dependent volume decline, it is unlikely that a common intervention for BDNF delivery would resolve age-related neuronal challenges in all regions. Hence, future investigations need to prioritize elucidating the complex mechanisms underlying BDNF function in the aging brain, particularly its role in enhancing resilience to aging-related cognitive decline and neurodegenerative disorders. Furthermore, researchers should delve into exploring novel therapeutic interventions that target BDNF pathways to promote resilience in older adults. Such interventions may include pharmacological agents and the development of small molecule compounds or biologics that specifically target BDNF receptors or signaling pathways to enhance BDNF levels or activity in the aging brain. For instance, pharmacological interventions targeting the MAPK/ERK pathways activated by BDNF/TrkB signaling could present new opportunities for developing therapeutic strategies aimed at enhancing neuronal resilience in the elderly. This can be achieved by upregulating anti-apoptotic proteins within the Bcl-2 family and downregulating pro-apoptotic factors such as Bax. Also, the activation of Ras, which triggers the sequential activation of the Ras/Raf/MEK/ERK signaling cascade, may further contribute to the inhibition of apoptotic signaling by suppressing pro-apoptotic proteins within the Bcl-2 family. Accordingly, the modulation of Bax/Bcl-2 signaling, which plays a central role in regulating neuronal apoptotic dynamics [267, 268] by BDNF/TrkB signaling and the MAPK/ERK pathways can potentially enhance neuronal survival and resilience. This modulation may help to fine-tune the balance between pro-apoptotic and anti-apoptotic factors, thus mitigating neurodegenerative processes and promoting cellular longevity. It was previously suggested that the neuroprotective action of BDNF could be a potential mechanism for counterregulating Bax/Bcl-2 proteins within the ischemic penumbra following focal ischemia [204].

**BOX 4:** *BDNF and its Synergy with other Biological Modulators in Aging* The brain is a complex system in which the synergy between its components is fundamental to its systemic function. BDNF synergizes with various molecules and compounds to promote brain health. These interactions can be leveraged to develop strategies for protecting neural integrity, preserving cognitive function and enhancing resilience in the elderly. Notable synergistic processes that may contribute to BDNF’s neuroprotective effects during aging include its cooperation with insulin, serotonin and oxytocin, antioxidants, and exercise-induced molecules. For instance, BDNF interacts with insulin signaling pathways to enhance glucose metabolism and protect neurons [287]. Because, insulin resistance, a common concern in aging, (e.g., brain-specific insulin signaling deficiencies in the early stages of AD pathogenesis, [288]) is linked to cognitive decline, BDNF interaction with insulin is crucial for maintaining energy metabolism and cognitive function. Also, BDNF and serotonin are involved in a complex biological communication; serotonergic signaling pathways modulate BDNF expression, and BDNF, in turn, regulates the development and function of serotonergic neurons [[289], see also [65] for more discussion]. Serotonin can also enhance the expression of BDNF in specific brain regions, such as the hippocampus [290]. Changes in BDNF levels during aging can also be hypothesized to impact synaptic function and compromise oxytocin-mediated synaptic plasticity [291], a condition that may lead to alterations in social behaviour and cognitive function. The interaction between BDNF and serotonin and oxytocin, therefore, supports neurogenesis and helps mitigate social disintegration and emotional disturbances experienced by older individuals and age-related cognitive decline. Furthermore, antioxidants, such as flavonoids and polyphenol, can protect neurons from oxidative stress and ROS while also increasing BDNF levels through both the ERK/CREB/BDNF and PI3K/Akt pathways [292]. Antioxidants such flavonoids [293] and polyphenols [294] support BDNF's role in promoting neuronal survival, thus enhancing its overall efficacy in preserving brain function during aging. An alternative way to increase BDNF levels in the aging brain is physical exercise [295-297], which is accompanied by elevated levels of irisin, an exercise-induced myokine that are primarily released by muscles in response to physical exercise [295, 298]. This synergistic relationship between BDNF and irisin highlights the importance of physical activity in maintaining brain health during aging. The synergistic interactions of BDNF with various biological modulators play a crucial role in maintaining neural integrity and cognitive function during aging. More importantly, these interactions offer significant potential for developing promising strategies to enhance brain resilience in the elderly.

Lifestyle modifications such as regular exercise (BOX 4), dietary changes, cognitive training or other lifestyle enrichments may also impact BDNF expression and function, thus promoting resilience against aging-related complications. Additionally, future investigations may need to evaluate the efficacy of nutraceuticals (dietary supplements and natural compounds) with known neurotrophic properties, such as polyphenols, omega-3 fatty acids, or flavonoids, in promoting BDNF expression and neuroplasticity in the aging brain. Genetic and epigenetic approaches to BDNF signaling and resilience can explore the feasibility of gene-based interventions, including viral vector delivery or CRISPR/Cas9 gene editing, to enhance BDNF expression or signaling in the brain as potential therapeutic strategies for improving resilience in aging. Furthermore, the potential of stem cell-based approaches, such as neural stem cell transplantation or induced pluripotent stem cell-derived neuronal grafts, has recently been suggested to promote BDNF production and neuroregeneration in the aging brain [269], thus enhancing resilience against aging-related neurodegeneration. However, it is important to note that while exerting influence on neural networks across various brain regions, BDNF may elicit distinct effects in specific brain areas. Therefore, interventions targeting a global elevation of BDNF in the brain may result in diminished efficacy compared to more localized interventions. In addition to these interventions, longitudinal studies examining the effects of BDNF modulation on cognitive resilience across different stages of aging are warranted to develop personalized interventions for optimizing brain health and resilience in the elderly population. Strategies for lowering age-dependent brain complications and improving resilience may emerge as high priorities for incorporation into social engineering interventions and aging health policies.

## References

[1] Goodman-Palmer D, Ferriolli E, Gordon AL, Greig C, Hirschhorn LR, Ogunyemi AO, et al. (2023). Health and wellbeing of older people in LMICs: a call for research-informed decision making. Lancet Glob Health, 11:e191-e192. 10.1016/S2214-109X(22)00546-0 36669801

[2] Chen C, Goldman DP, Zissimopoulos J, Rowe JW (2018). Multidimensional comparison of countries' adaptation to societal aging. Proc Natl Acad Sci U S A, 115:9169-9174. 10.1073/pnas.1806260115 PMC6140517 30154160

[3] Poganik JR, Gladyshev VN (2023). We need to shift the focus of aging research to aging itself. Proc Natl Acad Sci U S A, 120:e2307449120. 10.1073/pnas.2307449120 PMC10500180 37682890

[4] Mattson MP, Arumugam TV (2018). Hallmarks of Brain Aging: Adaptive and Pathological Modification by Metabolic States. Cell Metab, 27:1176-1199. 10.1016/j.cmet.2018.05.011 PMC6039826 29874566

[5] Bieri G, Schroer AB, Villeda SA (2023). Blood-to-brain communication in aging and rejuvenation. Nat Neurosci, 26:379-393. 10.1038/s41593-022-01238-8 36646876

[6] Molinari C, Morsanuto V, Ruga S, Notte F, Farghali M, Galla R, et al. (2020). The Role of BDNF on Aging-Modulation Markers. Brain Sci, 10. 10.3390/brainsci10050285 PMC7287884 32397504

[7] Cevenini E, Invidia L, Lescai F, Salvioli S, Tieri P, Castellani G, et al. (2008). Human models of aging and longevity. Expert Opin Biol Ther, 8:1393-1405. 10.1517/14712598.8.9.1393 18694357

[8] López-Otín C, Blasco MA, Partridge L, Serrano M, Kroemer G (2023). Hallmarks of aging: An expanding universe. Cell, 186:243-278. 10.1016/j.cell.2022.11.001 36599349

[9] Kennedy BK, Berger SL, Brunet A, Campisi J, Cuervo AM, Epel ES, et al. (2014). Geroscience: linking aging to chronic disease. Cell, 159:709-713. 10.1016/j.cell.2014.10.039 PMC4852871 25417146

[10] Setton R, Mwilambwe-Tshilobo L, Sheldon S, Turner GR, Spreng RN (2022). Hippocampus and temporal pole functional connectivity is associated with age and individual differences in autobiographical memory. Proc Natl Acad Sci U S A, 119:e2203039119. 10.1073/pnas.2203039119 PMC9564102 36191210

[11] Zheng L, Gao Z, Doner S, Oyao A, Forloines M, Grilli MD, et al. (2023). Hippocampal contributions to novel spatial learning are both age-related and age-invariant. Proc Natl Acad Sci U S A, 120:e2307884120. 10.1073/pnas.2307884120 PMC10723126 38055735

[12] Markov NT, Lindbergh CA, Staffaroni AM, Perez K, Stevens M, Nguyen K, et al. (2022). Age-related brain atrophy is not a homogenous process: Different functional brain networks associate differentially with aging and blood factors. Proc Natl Acad Sci U S A, 119:e2207181119. 10.1073/pnas.2207181119 PMC9894212 36459652

[13] Chan MY, Park DC, Savalia NK, Petersen SE, Wig GS (2014). Decreased segregation of brain systems across the healthy adult lifespan. Proc Natl Acad Sci U S A, 111:E4997-5006. 10.1073/pnas.1415122111 PMC4246293 25368199

[14] Ewers M, Luan Y, Frontzkowski L, Neitzel J, Rubinski A, Dichgans M, et al. (2021). Segregation of functional networks is associated with cognitive resilience in Alzheimer's disease. Brain, 144:2176-2185. 10.1093/brain/awab112 PMC8370409 33725114

[15] Cole JH, Marioni RE, Harris SE, Deary IJ (2019). Brain age and other bodily 'ages': implications for neuropsychiatry. Mol Psychiatry, 24:266-281. 10.1038/s41380-018-0098-1 PMC6344374 29892055

[16] Li G, McLaughlin DW, Peskin CS (2024). A biochemical description of postsynaptic plasticity-with timescales ranging from milliseconds to seconds. Proc Natl Acad Sci U S A, 121:e2311709121. 10.1073/pnas.2311709121 PMC10873618 38324573

[17] López-Otín C, Kroemer G (2021). Hallmarks of Health. Cell, 184:33-63. 10.1016/j.cell.2020.11.034 33340459

[18] Heinrichs J-H (2023). Brain age Prediction and the Challenge of Biological Concepts of Aging. Neuroethics, 16:1-13.

[19] Cole JH, Ritchie SJ, Bastin ME, Valdés Hernández MC, Muñoz Maniega S, Royle N, et al. (2018). Brain age predicts mortality. Mol Psychiatry, 23:1385-1392. 10.1038/mp.2017.62 PMC5984097 28439103

[20] Tian YE, Cropley V, Maier AB, Lautenschlager NT, Breakspear M, Zalesky A (2023). Heterogeneous aging across multiple organ systems and prediction of chronic disease and mortality. Nat Med, 29:1221-1231. 10.1038/s41591-023-02296-6 37024597

[21] Khan R, Di Gesù CM, Lee J, McCullough LD (2024). The contribution of age-related changes in the gut-brain axis to neurological disorders. Gut Microbes, 16:2302801. 10.1080/19490976.2024.2302801 PMC10798364 38237031

[22] Ferrarelli LK (2022). Brain aging by gut AGE. Sci Signal, 15:eabq3005. 10.1126/scisignal.abq3005 35380878

[23] Promislow D, Anderson RM, Scheffer M, Crespi B, DeGregori J, Harris K, et al. (2022). Resilience integrates concepts in aging research. iScience, 25:104199. 10.1016/j.isci.2022.104199 PMC9044173 35494229

[24] Akil H, Nestler EJ (2023). The neurobiology of stress: Vulnerability, resilience, and major depression. Proc Natl Acad Sci U S A, 120:e2312662120. 10.1073/pnas.2312662120 PMC10710064 38011574

[25] Hofgaard LS, Nes RB, Røysamb E (2021). Introducing two types of psychological resilience with partly unique genetic and environmental sources. Sci Rep, 11:8624. 10.1038/s41598-021-87581-5 PMC8060303 33883571

[26] Hunter RG, Gray JD, McEwen BS (2018). The neuroscience of resilience. Journal of the Society for Social Work and Research, 9:305-339.

[27] Wu G, Feder A, Cohen H, Kim JJ, Calderon S, Charney DS, et al. (2013). Understanding resilience. Front Behav Neurosci, 7:10. 10.3389/fnbeh.2013.00010 PMC3573269 23422934

[28] Troy AS, Willroth EC, Shallcross AJ, Giuliani NR, Gross JJ, Mauss IB (2023). Psychological Resilience: An Affect-Regulation Framework. Annu Rev Psychol, 74:547-576. 10.1146/annurev-psych-020122-041854 PMC12009612 36103999

[29] Masten AS, Barnes AJ (2018). Resilience in Children: Developmental Perspectives. Children(Basel), 5. 10.3390/children5070098 PMC6069421 30018217

[30] Boyce WT, Ellis BJ (2005). Biological sensitivity to context: I. An evolutionary-developmental theory of the origins and functions of stress reactivity. Dev Psychopathol, 17:271-301. 10.1017/s0954579405050145 16761546

[31] McEwen BS, Wingfield JC (2010). What is in a name? Integrating homeostasis, allostasis and stress. Horm Behav, 57:105-111. 10.1016/j.yhbeh.2009.09.011 PMC2815096 19786032

[32] Klopack ET, Crimmins EM, Cole SW, Seeman TE, Carroll JE (2022). Social stressors associated with age-related T lymphocyte percentages in older US adults: Evidence from the US Health and Retirement Study. Proc Natl Acad Sci U S A, 119:e2202780119. 10.1073/pnas.2202780119 PMC9231620 35696572

[33] McEwen BS (1998). Stress, adaptation, and disease. Allostasis and allostatic load. Ann N Y Acad Sci, 840:33-44. 10.1111/j.1749-6632.1998.tb09546.x 9629234

[34] Olson DM, Severson EM, Verstraeten BS, Ng JW, McCreary JK, Metz GA (2015). Allostatic Load and Preterm Birth. Int J Mol Sci, 16:29856-29874. 10.3390/ijms161226209 PMC4691152 26694355

[35] Guan Y, Shen J, Lu J, Fuemmeler BF, Shock LS, Zhao H (2023). Association between allostatic load and breast cancer risk: a cohort study. Breast Cancer Res, 25:155. 10.1186/s13058-023-01754-w PMC10729373 38115125

[36] Thomson EM, Walker M, Halverson-Duncan B (2024). Harmonizing the assessment of allostatic load across cycles of the Canadian Health Measures Survey: Variable selection and calculation method. Health Rep, 35:16-25. 10.25318/82-003-x202400500002-eng 38758724

[37] McCreary JK, Erickson ZT, Paxman E, Kiss D, Montina T, Olson DM, et al. (2019). The rat cumulative allostatic load measure (rCALM): a new translational assessment of the burden of stress. Environ Epigenet, 5:dvz005. 10.1093/eep/dvz005 PMC6500369 31065381

[38] Reynolds TAA, Mirela; Falkenberg, Erin A.; Awosoga Oluwagbohunmi A. and Metz Gerlinde A.S. (2024). Maternal Allostatic Load as a Predictor of Adverse Pregnancy Outcomes and Offspring Development in the F3 Generation: Evidence from a Rat Model of Transgenerational and Multigenerational Stress. Archives of Clinical and Biomedical Research., 8:153-166.

[39] Kabacik S, Lowe D, Fransen L, Leonard M, Ang S-L, Whiteman C, et al. (2022). The relationship between epigenetic age and the hallmarks of aging in human cells. Nature Aging, 2:484-493. 10.1038/s43587-022-00220-0 PMC10077971 37034474

[40] Moqri M, Herzog C, Poganik JR, Justice J, Belsky DW, Higgins-Chen A, et al. (2023). Biomarkers of aging for the identification and evaluation of longevity interventions. Cell, 186:3758-3775. 10.1016/j.cell.2023.08.003 PMC11088934 37657418

[41] Horvath S, Raj K (2018). DNA methylation-based biomarkers and the epigenetic clock theory of ageing. Nat Rev Genet, 19:371-384. 10.1038/s41576-018-0004-3 29643443

[42] Faraji J, Metz GAS (2023). Toward reframing brain-social dynamics: current assumptions and future challenges. Front Psychiatry, 14:1211442. 10.3389/fpsyt.2023.1211442 PMC10359502 37484686

[43] Poplawski J, Radmilovic A, Montina TD, Metz GAS (2020). Cardiorenal metabolic biomarkers link early life stress to risk of non-communicable diseases and adverse mental health outcomes. Sci Rep, 10:13295. 10.1038/s41598-020-69866-3 PMC7413400 32764629

[44] Phua DY, Chen H, Yap F, Chong YS, Gluckman PD, Broekman BFP, et al. (2023). Allostatic load in children: The cost of empathic concern. Proc Natl Acad Sci U S A, 120:e2217769120. 10.1073/pnas.2217769120 PMC10523447 37725642

[45] Moffitt TE, Arseneault L, Belsky D, Dickson N, Hancox RJ, Harrington H, et al. (2011). A gradient of childhood self-control predicts health, wealth, and public safety. Proc Natl Acad Sci U S A, 108:2693-2698. 10.1073/pnas.1010076108 PMC3041102 21262822

[46] Faraji J, Metz GAS (2021). Aging, Social Distancing, and COVID-19 Risk: Who is more Vulnerable and Why? Aging Dis, 12:1624-1643. 10.14336/AD.2021.0319 PMC8460299 34631211

[47] Aiello A, Farzaneh F, Candore G, Caruso C, Davinelli S, Gambino CM, et al. (2019). Immunosenescence and Its Hallmarks: How to Oppose Aging Strategically? A Review of Potential Options for Therapeutic Intervention. Front Immunol, 10:2247. 10.3389/fimmu.2019.02247 PMC6773825 31608061

[48] Sidler C, Wóycicki R, Ilnytskyy Y, Metz G, Kovalchuk I, Kovalchuk O (2013). Immunosenescence is associated with altered gene expression and epigenetic regulation in primary and secondary immune organs. Front Genet, 4:211. 10.3389/fgene.2013.00211 PMC3798808 24151501

[49] Fulop T, Larbi A, Dupuis G, Le Page A, Frost EH, Cohen AA, et al. (2017). Immunosenescence and Inflamm-Aging As Two Sides of the Same Coin: Friends or Foes? Front Immunol, 8:1960. 10.3389/fimmu.2017.01960 PMC5767595 29375577

[50] Franceschi C, Bonafè M, Valensin S, Olivieri F, De Luca M, Ottaviani E, et al. (2000). Inflamm-aging. An evolutionary perspective on immunosenescence. Ann N Y Acad Sci, 908:244-254. 10.1111/j.1749-6632.2000.tb06651.x 10911963

[51] Ahuja SK, Manoharan MS, Lee GC, McKinnon LR, Meunier JA, Steri M, et al. (2023). Immune resilience despite inflammatory stress promotes longevity and favorable health outcomes including resistance to infection. Nat Commun, 14:3286. 10.1038/s41467-023-38238-6 PMC10264401 37311745

[52] Maggio M, Guralnik JM, Longo DL, Ferrucci L (2006). Interleukin-6 in aging and chronic disease: a magnificent pathway. J Gerontol A Biol Sci Med Sci, 61:575-584. 10.1093/gerona/61.6.575 PMC2645627 16799139

[53] Koelman L, Pivovarova-Ramich O, Pfeiffer AFH, Grune T, Aleksandrova K (2019). Cytokines for evaluation of chronic inflammatory status in ageing research: reliability and phenotypic characterisation. Immun Ageing, 16:11. 10.1186/s12979-019-0151-1 PMC6530020 31139232

[54] Del Giudice M, Gangestad SW (2018). Rethinking IL-6 and CRP: Why they are more than inflammatory biomarkers, and why it matters. Brain Behav Immun, 70:61-75. 10.1016/j.bbi.2018.02.013 29499302

[55] Almeida RD, Manadas BJ, Melo CV, Gomes JR, Mendes CS, Grãos MM, et al. (2005). Neuroprotection by BDNF against glutamate-induced apoptotic cell death is mediated by ERK and PI3-kinase pathways. Cell Death Differ, 12:1329-1343. 10.1038/sj.cdd.4401662 15905876

[56] Chen SD, Wu CL, Hwang WC, Yang DI (2017). More Insight into BDNF against Neurodegeneration: Anti-Apoptosis, Anti-Oxidation, and Suppression of Autophagy. Int J Mol Sci, 18. 10.3390/ijms18030545 PMC5372561 28273832

[57] Minuzzi LG, Seelaender M, Silva BSA, Cunha E, Deus MC, Vasconcellos FTF, et al. (2021). COVID-19 Outcome Relates With Circulating BDNF, According to Patient Adiposity and Age. Front Nutr, 8:784429. 10.3389/fnut.2021.784429 PMC8704131 34957187

[58] Biamonte F, Re A, Balzamino BO, Ciasca G, Santucci D, Napodano C, et al. (2022). Circulating and Salivary NGF and BDNF Levels in SARS-CoV-2 Infection: Potential Predictor Biomarkers of COVID-19 Disease-Preliminary Data. J Pers Med, 12. 10.3390/jpm12111877 PMC9697501 36579579

[59] Shafiee A, Seighali N, Teymouri Athar M, Abdollahi AK, Jafarabady K, Bakhtiyari M (2024). Levels of brain-derived neurotrophic factor (BDNF) among patients with COVID-19: a systematic review and meta-analysis. Eur Arch Psychiatry Clin Neurosci, 274:1137-1152. 10.1007/s00406-023-01681-z 37646849

[60] Bellavance MA, Rivest S (2014). The HPA - Immune Axis and the Immunomodulatory Actions of Glucocorticoids in the Brain. Front Immunol, 5:136. 10.3389/fimmu.2014.00136 PMC3978367 24744759

[61] Sapolsky RM, Krey LC, McEwen BS (1986). The neuroendocrinology of stress and aging: the glucocorticoid cascade hypothesis. Endocr Rev, 7:284-301. 10.1210/edrv-7-3-284 3527687

[62] Tapia-Arancibia L, Rage F, Givalois L, Arancibia S (2004). Physiology of BDNF: focus on hypothalamic function. Front Neuroendocrinol, 25:77-107. 10.1016/j.yfrne.2004.04.001 15571756

[63] Naert G, Zussy C, Tran Van Ba C, Chevallier N, Tang YP, Maurice T, et al. (2015). Involvement of Endogenous Brain-Derived Neurotrophic Factor in Hypothalamic-Pituitary-Adrenal Axis Activity. J Neuroendocrinol, 27:850-860. 10.1111/jne.12324 26388293

[64] Faraji J, Karimi M, Soltanpour N, Rouhzadeh Z, Roudaki S, Hosseini SA, et al. (2018). Intergenerational Sex-Specific Transmission of Maternal Social Experience. Sci Rep, 8:10529. 10.1038/s41598-018-28729-8 PMC6043535 30002484

[65] Correia AS, Cardoso A, Vale N (2023). BDNF Unveiled: Exploring Its Role in Major Depression Disorder Serotonergic Imbalance and Associated Stress Conditions. Pharmaceutics, 15. 10.3390/pharmaceutics15082081 PMC10457827 37631295

[66] Krüttgen A, Möller JC, Heymach JV Jr., Shooter EM (1998). Neurotrophins induce release of neurotrophins by the regulated secretory pathway. Proc Natl Acad Sci U S A, 95:9614-9619. 10.1073/pnas.95.16.9614 PMC21387 9689129

[67] Sochal M, Ditmer M, Binienda A, Tarasiuk A, Białasiewicz P, Turkiewicz S, et al. (2024). Interactions between neurotrophins, mood, and physical activity under the conditions of sleep deprivation. Transl Psychiatry, 14:158. 10.1038/s41398-024-02871-6 PMC10960007 38519465

[68] Fukuyama Y, Kubo M, Harada K (2024). Neurotrophic Natural Products. Prog Chem Org Nat Prod, 123:1-473. 10.1007/978-3-031-42422-9_1 38340248

[69] Boxy P, Nykjær A, Kisiswa L (2023). Building better brains: the pleiotropic function of neurotrophic factors in postnatal cerebellar development. Front Mol Neurosci, 16:1181397. 10.3389/fnmol.2023.1181397 PMC10213292 37251644

[70] Metz GA, Faraji J. 2009. Growth inhibitory molecules in nervous system development and regeneration. In Encyclopedia of Neuroscience MD B., N H., and U W., editors. Heidelberg: Springer Verlag. 1785-1789

[71] Jin W (2020). Regulation of BDNF-TrkB Signaling and Potential Therapeutic Strategies for Parkinson's Disease. J Clin Med, 9. 10.3390/jcm9010257 PMC7019526 31963575

[72] Eu WZ, Chen Y-J, Chen W-T, Wu K-Y, Tsai C-Y, Cheng S-J, et al. (2021). The effect of nerve growth factor on supporting spatial memory depends upon hippocampal cholinergic innervation. Translational Psychiatry, 11:162. 10.1038/s41398-021-01280-3 PMC7961060 33723225

[73] Do Carmo S, Kannel B, Cuello AC (2021). The Nerve Growth Factor Metabolic Pathway Dysregulation as Cause of Alzheimer's Cholinergic Atrophy. Cells, 11. 10.3390/cells11010016 PMC8750266 35011577

[74] Counts SE, Mufson EJ (2005). The Role of Nerve Growth Factor Receptors in Cholinergic Basal Forebrain Degeneration in Prodromal Alzheimer Disease. Journal of Neuropathology & Experimental Neurology, 64:263-272. 10.1093/jnen/64.4.263 15835262

[75] Loch AA, Pinto MTC, Andrade JC, de Jesus LP, de Medeiros MW, Haddad NM, et al. (2023). Plasma levels of neurotrophin 4/5, NGF and pro-BDNF influence transition to mental disorders in a sample of individuals at ultra-high risk for psychosis. Psychiatry Res, 327:115402. 10.1016/j.psychres.2023.115402 37544089

[76] Tonyan S, Pospelova M, Krasnikova V, Fionik O, Alekseeva T, Samochernykh K, et al. (2023). Neurotrophin-3 (NT-3) as a Potential Biomarker of the Peripheral Nervous System Damage Following Breast Cancer Treatment. Pathophysiology, 30:110-122. 10.3390/pathophysiology30020010 PMC10123681 37092524

[77] Klein R, Silos-Santiago I, Smeyne RJ, Lira SA, Brambilla R, Bryant S, et al. (1994). Disruption of the neurotrophin-3 receptor gene trkC eliminates la muscle afferents and results in abnormal movements. Nature, 368:249-251. 10.1038/368249a0 8145824

[78] Delezie J, Weihrauch M, Maier G, Tejero R, Ham DJ, Gill JF, et al. (2019). BDNF is a mediator of glycolytic fiber-type specification in mouse skeletal muscle. Proc Natl Acad Sci U S A, 116:16111-16120. 10.1073/pnas.1900544116 PMC6690026 31320589

[79] Fischer W, Sirevaag A, Wiegand SJ, Lindsay RM, Björklund A (1994). Reversal of spatial memory impairments in aged rats by nerve growth factor and neurotrophins 3 and 4/5 but not by brain-derived neurotrophic factor. Proc Natl Acad Sci U S A, 91:8607-8611. 10.1073/pnas.91.18.8607 PMC44655 8078930

[80] Friedman W. 2012. Chapter 29 - Growth Factors. In Basic Neurochemistry (Eighth Edition). Brady S.T., Siegel G.J., Albers R.W., and Price D.L., editors. New York: Academic Press. 546-557.

[81] Torres-Cruz FM, César Vivar-Cortés I, Moran I, Mendoza E, Gómez-Pineda V, García-Sierra F, et al. (2019). NT-4/5 antagonizes the BDNF modulation of corticostriatal transmission: Role of the TrkB.T1 receptor. CNS Neurosci Ther, 25:621-631. 10.1111/cns.13091 PMC6488875 30666798

[82] Barde YA, Edgar D, Thoenen H (1982). Purification of a new neurotrophic factor from mammalian brain. Embo j, 1:549-553. 10.1002/j.1460-2075.1982.tb01207.x PMC553086 7188352

[83] Kandel ER. 2006. In search of memory: The emergence of a new science of mind. New York, NY, US: W W Norton & Co. xv, 510-xv, 510 pp.

[84] Wang CS, McCarthy CI, Guzikowski NJ, Kavalali ET, Monteggia LM (2024). Brain-derived neurotrophic factor scales presynaptic calcium transients to modulate excitatory neurotransmission. Proc Natl Acad Sci U S A, 121:e2303664121. 10.1073/pnas.2303664121 PMC11047077 38621124

[85] Miller KM, Mercado NM, Sortwell CE (2021). Synucleinopathy-associated pathogenesis in Parkinson's disease and the potential for brain-derived neurotrophic factor. NPJ Parkinsons Dis, 7:35. 10.1038/s41531-021-00179-6 PMC8041900 33846345

[86] Miranda M, Morici JF, Zanoni MB, Bekinschtein P (2019). Brain-Derived Neurotrophic Factor: A Key Molecule for Memory in the Healthy and the Pathological Brain. Front Cell Neurosci, 13:363. 10.3389/fncel.2019.00363 PMC6692714 31440144

[87] Je HS, Yang F, Ji Y, Potluri S, Fu XQ, Luo ZG, et al. (2013). ProBDNF and mature BDNF as punishment and reward signals for synapse elimination at mouse neuromuscular junctions. J Neurosci, 33:9957-9962. 10.1523/JNEUROSCI.0163-13.2013 PMC3682390 23761891

[88] Yang B, Ren Q, Zhang JC, Chen QX, Hashimoto K (2017). Altered expression of BDNF, BDNF pro-peptide and their precursor proBDNF in brain and liver tissues from psychiatric disorders: rethinking the brain-liver axis. Transl Psychiatry, 7:e1128. 10.1038/tp.2017.95 PMC5534963 28509900

[89] Je HS, Yang F, Ji Y, Nagappan G, Hempstead BL, Lu B (2012). Role of pro-brain-derived neurotrophic factor (proBDNF) to mature BDNF conversion in activity-dependent competition at developing neuromuscular synapses. Proc Natl Acad Sci U S A, 109:15924-15929. 10.1073/pnas.1207767109 PMC3465384 23019376

[90] Woo NH, Teng HK, Siao CJ, Chiaruttini C, Pang PT, Milner TA, et al. (2005). Activation of p75NTR by proBDNF facilitates hippocampal long-term depression. Nat Neurosci, 8:1069-1077. 10.1038/nn1510 16025106

[91] Friedman WJ (2010). Proneurotrophins, seizures, and neuronal apoptosis. Neuroscientist, 16:244-252. 10.1177/1073858409349903 PMC2956880 20360602

[92] Nagahara AH, Tuszynski MH (2011). Potential therapeutic uses of BDNF in neurological and psychiatric disorders. Nat Rev Drug Discov, 10:209-219. 10.1038/nrd3366 21358740

[93] Elhadidy ME, Kilany A, Gebril OH, Nashaat NH, Zeidan HM, Elsaied A, et al. (2023). BDNF Val66Met Polymorphism: Suggested Genetic Involvement in Some Children with Learning Disorder. J Mol Neurosci, 73:39-46. 10.1007/s12031-022-02095-7 PMC9894953 36550387

[94] Kennedy KM, Reese ED, Horn MM, Sizemore AN, Unni AK, Meerbrey ME, et al. (2015). BDNF val66met polymorphism affects aging of multiple types of memory. Brain Res, 1612:104-117. 10.1016/j.brainres.2014.09.044 PMC4377126 25264352

[95] Puri R, Hinder MR, Fujiyama H, Gomez R, Carson RG, Summers JJ (2015). Duration-dependent effects of the BDNF Val66Met polymorphism on anodal tDCS induced motor cortex plasticity in older adults: a group and individual perspective. Front Aging Neurosci, 7:107. 10.3389/fnagi.2015.00107 PMC4456583 26097454

[96] Abellaneda-Pérez K, Martin-Trias P, Cassé-Perrot C, Vaqué-Alcázar L, Lanteaume L, Solana E, et al. (2022). BDNF Val66Met gene polymorphism modulates brain activity following rTMS-induced memory impairment. Scientific Reports, 12:176. 10.1038/s41598-021-04175-x PMC8741781 34997117

[97] Privodnova EY, Volf NV (2023). Association of the Brain-Derived Neurotrophic Factor Gene (BDNF) Val66Met Polymorphism with Individual Alpha Peak Frequency and Alpha Power in Adults. Human Physiology, 49:357-363.

[98] Brown DT, Vickers JC, Stuart KE, Cechova K, Ward DD (2020). The BDNF Val66Met Polymorphism Modulates Resilience of Neurological Functioning to Brain Ageing and Dementia: A Narrative Review. Brain Sci, 10. 10.3390/brainsci10040195 PMC7226504 32218234

[99] von Bohlen und Halbach O (2010). Involvement of BDNF in age-dependent alterations in the hippocampus. Front Aging Neurosci, 2. 10.3389/fnagi.2010.00036 PMC2952461 20941325

[100] McEwen BS (2012). Brain on stress: how the social environment gets under the skin. Proc Natl Acad Sci U S A, 109 Suppl 2:17180-17185. 10.1073/pnas.1121254109 PMC3477378 23045648

[101] Ling E, Nemesh J, Goldman M, Kamitaki N, Reed N, Handsaker RE, et al. (2024). A concerted neuron-astrocyte program declines in ageing and schizophrenia. Nature. 10.1038/s41586-024-07109-5 PMC10954558 38448582

[102] Stanford WC, Mucha PJ, Dayan E (2022). A robust core architecture of functional brain networks supports topological resilience and cognitive performance in middle- and old-aged adults. Proc Natl Acad Sci U S A, 119:e2203682119. 10.1073/pnas.2203682119 PMC9636938 36282912

[103] Montaron MF, Charrier V, Blin N, Garcia P, Abrous DN (2020). Responsiveness of dentate neurons generated throughout adult life is associated with resilience to cognitive aging. Aging Cell, 19:e13161. 10.1111/acel.13161 PMC7431828 32599664

[104] Wyss-Coray T (2016). Ageing, neurodegeneration and brain rejuvenation. Nature, 539:180-186. 10.1038/nature20411 PMC5172605 27830812

[105] Kampmann M (2024). Molecular and cellular mechanisms of selective vulnerability in neurodegenerative diseases. Nature Reviews Neuroscience. 10.1038/s41583-024-00806-0 38575768

[106] Cuanalo-Contreras K, Schulz J, Mukherjee A, Park KW, Armijo E, Soto C (2022). Extensive accumulation of misfolded protein aggregates during natural aging and senescence. Front Aging Neurosci, 14:1090109. 10.3389/fnagi.2022.1090109 PMC9909609 36778589

[107] Ciechanover A, Schwartz AL (1998). The ubiquitin-proteasome pathway: the complexity and myriad functions of proteins death. Proc Natl Acad Sci U S A, 95:2727-2730. 10.1073/pnas.95.6.2727 PMC34259 9501156

[108] Gong B, Radulovic M, Figueiredo-Pereira ME, Cardozo C (2016). The Ubiquitin-Proteasome System: Potential Therapeutic Targets for Alzheimer's Disease and Spinal Cord Injury. Front Mol Neurosci, 9:4. 10.3389/fnmol.2016.00004 PMC4727241 26858599

[109] Finkbeiner S (2020). The Autophagy Lysosomal Pathway and Neurodegeneration. Cold Spring Harb Perspect Biol, 12. 10.1101/cshperspect.a033993 PMC6773515 30936119

[110] Li M, Tripathi-Giesgen I, Schulman BA, Baumeister W, Wilfling F (2023). In situ snapshots along a mammalian selective autophagy pathway. Proc Natl Acad Sci U S A, 120:e2221712120. 10.1073/pnas.2221712120 PMC10041112 36917659

[111] Vilchez D, Saez I, Dillin A (2014). The role of protein clearance mechanisms in organismal ageing and age-related diseases. Nature Communications, 5:5659. 10.1038/ncomms6659 25482515

[112] Hetz C (2021). Adapting the proteostasis capacity to sustain brain healthspan. Cell, 184:1545-1560. 10.1016/j.cell.2021.02.007 33691137

[113] Morgan GR, Carlyle BC (2024). Interrogation of the human cortical peptidome uncovers cell-type specific signatures of cognitive resilience against Alzheimer’s disease. Scientific Reports, 14:7161. 10.1038/s41598-024-57104-z PMC10966065 38531951

[114] Rapoport M, Dawson HN, Binder LI, Vitek MP, Ferreira A (2002). Tau is essential to beta -amyloid-induced neurotoxicity. Proc Natl Acad Sci U S A, 99:6364-6369. 10.1073/pnas.092136199 PMC122954 11959919

[115] Bloom GS (2014). Amyloid-β and tau: the trigger and bullet in Alzheimer disease pathogenesis. JAMA Neurol, 71:505-508. 10.1001/jamaneurol.2013.5847 PMC12908160 24493463

[116] Gitler AD, Bevis BJ, Shorter J, Strathearn KE, Hamamichi S, Su LJ, et al. (2008). The Parkinson's disease protein alpha-synuclein disrupts cellular Rab homeostasis. Proc Natl Acad Sci U S A, 105:145-150. 10.1073/pnas.0710685105 PMC2224176 18162536

[117] Tong Y, Zhang P, Yang X, Liu X, Zhang J, Grudniewska M, et al. (2024). Decreasing the intrinsically disordered protein α-synuclein levels by targeting its structured mRNA with a ribonuclease-targeting chimera. Proc Natl Acad Sci U S A, 121:e2306682120. 10.1073/pnas.2306682120 PMC10786272 38181056

[118] Vauleon S, Schutz K, Massonnet B, Gruben N, Manchester M, Buehler A, et al. (2023). Quantifying mutant huntingtin protein in human cerebrospinal fluid to support the development of huntingtin-lowering therapies. Sci Rep, 13:5332. 10.1038/s41598-023-32630-4 PMC10067853 37005488

[119] Parkin GM, Corey-Bloom J, Snell C, Smith H, Laurenza A, Daldin M, et al. (2023). Salivary Huntingtin protein is uniquely associated with clinical features of Huntington's disease. Sci Rep, 13:1034. 10.1038/s41598-023-28019-y PMC9852574 36658243

[120] Xiang J, Wang ZH, Ahn EH, Liu X, Yu SP, Manfredsson FP, et al. (2019). Delta-secretase-cleaved Tau antagonizes TrkB neurotrophic signalings, mediating Alzheimer's disease pathologies. Proc Natl Acad Sci U S A, 116:9094-9102. 10.1073/pnas.1901348116 PMC6500177 31004063

[121] Li H, Cao Y, Ye J, Yang Z, Chen Q, Liu X, et al. (2023). Engineering brain-derived neurotrophic factor mRNA delivery for the treatment of Alzheimer’s disease. Chemical Engineering Journal, 466:143152.

[122] Jiao SS, Shen LL, Zhu C, Bu XL, Liu YH, Liu CH, et al. (2016). Brain-derived neurotrophic factor protects against tau-related neurodegeneration of Alzheimer's disease. Transl Psychiatry, 6:e907. 10.1038/tp.2016.186 PMC5315549 27701410

[123] Kaspar BK, Vissel B, Bengoechea T, Crone S, Randolph-Moore L, Muller R, et al. (2002). Adeno-associated virus effectively mediates conditional gene modification in the brain. Proc Natl Acad Sci U S A, 99:2320-2325. 10.1073/pnas.042678699 PMC122363 11842206

[124] Shen F, Kuo R, Milon-Camus M, Han Z, Jiang L, Young WL, et al. (2013). Intravenous delivery of adeno-associated viral vector serotype 9 mediates effective gene expression in ischemic stroke lesion and brain angiogenic foci. Stroke, 44:252-254. 10.1161/STROKEAHA.112.662965 PMC3531817 23250995

[125] Arora S, Sharma D, Singh J (2020). GLUT-1: An Effective Target To Deliver Brain-Derived Neurotrophic Factor Gene Across the Blood Brain Barrier. ACS Chem Neurosci, 11:1620-1633. 10.1021/acschemneuro.0c00076 32352752

[126] Castello NA, Green KN, LaFerla FM (2012). Genetic knockdown of brain-derived neurotrophic factor in 3xTg-AD mice does not alter Aβ or tau pathology. PLoS One, 7:e39566. 10.1371/journal.pone.0039566 PMC3411687 22870188

[127] Gao L, Zhang Y, Sterling K, Song W (2022). Brain-derived neurotrophic factor in Alzheimer's disease and its pharmaceutical potential. Transl Neurodegener, 11:4. 10.1186/s40035-022-00279-0 PMC8796548 35090576

[128] Howells DW, Porritt MJ, Wong JY, Batchelor PE, Kalnins R, Hughes AJ, et al. (2000). Reduced BDNF mRNA expression in the Parkinson's disease substantia nigra. Exp Neurol, 166:127-135. 10.1006/exnr.2000.7483 11031089

[129] Cao Q, Luo S, Yao W, Qu Y, Wang N, Hong J, et al. (2022). Suppression of abnormal α-synuclein expression by activation of BDNF transcription ameliorates Parkinson's disease-like pathology. Mol Ther Nucleic Acids, 29:1-15. 10.1016/j.omtn.2022.05.037 PMC9207554 35784012

[130] Fang F, Yang W, Florio JB, Rockenstein E, Spencer B, Orain XM, et al. (2017). Synuclein impairs trafficking and signaling of BDNF in a mouse model of Parkinson's disease. Sci Rep, 7:3868. 10.1038/s41598-017-04232-4 PMC5478665 28634349

[131] Wang YC, Feng GY, Xia QJ, Hu Y, Xu Y, Xiong LL, et al. (2016). Knockdown of α-synuclein in cerebral cortex improves neural behavior associated with apoptotic inhibition and neurotrophin expression in spinal cord transected rats. Apoptosis, 21:404-420. 10.1007/s10495-016-1218-5 26822976

[132] Kang SS, Zhang Z, Liu X, Manfredsson FP, Benskey MJ, Cao X, et al. (2017). TrkB neurotrophic activities are blocked by α-synuclein, triggering dopaminergic cell death in Parkinson's disease. Proc Natl Acad Sci U S A, 114:10773-10778. 10.1073/pnas.1713969114 PMC5635931 28923922

[133] Espay AJ, Schwarzschild MA, Tanner CM, Fernandez HH, Simon DK, Leverenz JB, et al. (2017). Biomarker-driven phenotyping in Parkinson's disease: A translational missing link in disease-modifying clinical trials. Mov Disord, 32:319-324. 10.1002/mds.26913 PMC5359057 28233927

[134] Lev N, Melamed E, Offen D (2003). Apoptosis and Parkinson's disease. Prog Neuropsychopharmacol Biol Psychiatry, 27:245-250. 10.1016/S0278-5846(03)00019-8 12657363

[135] Jiang T, Yu JT, Zhu XC, Wang HF, Tan MS, Cao L, et al. (2014). Acute metformin preconditioning confers neuroprotection against focal cerebral ischaemia by pre-activation of AMPK-dependent autophagy. Br J Pharmacol, 171:3146-3157. 10.1111/bph.12655 PMC4080970 24611741

[136] Zia A, Pourbagher-Shahri AM, Farkhondeh T, Samarghandian S (2021). Molecular and cellular pathways contributing to brain aging. Behav Brain Funct, 17:6. 10.1186/s12993-021-00179-9 PMC8199306 34118939

[137] Harman D (1992). Free radical theory of aging: history. Exs, 62:1-10. 10.1007/978-3-0348-7460-1_1 1450577

[138] Knox EG, Aburto MR, Clarke G, Cryan JF, O'Driscoll CM (2022). The blood-brain barrier in aging and neurodegeneration. Mol Psychiatry, 27:2659-2673. 10.1038/s41380-022-01511-z PMC9156404 35361905

[139] Cheng A, Wan R, Yang JL, Kamimura N, Son TG, Ouyang X, et al. (2012). Involvement of PGC-1α in the formation and maintenance of neuronal dendritic spines. Nat Commun, 3:1250. 10.1038/ncomms2238 PMC4091730 23212379

[140] Jęśko H, Wencel P, Strosznajder RP, Strosznajder JB (2017). Sirtuins and Their Roles in Brain Aging and Neurodegenerative Disorders. Neurochem Res, 42:876-890. 10.1007/s11064-016-2110-y PMC5357501 27882448

[141] Shen J, Xu L, Qu C, Sun H, Zhang J (2018). Resveratrol prevents cognitive deficits induced by chronic unpredictable mild stress: Sirt1/miR-134 signalling pathway regulates CREB/BDNF expression in hippocampus in vivo and in vitro. Behav Brain Res, 349:1-7. 10.1016/j.bbr.2018.04.050 29715537

[142] Caruso GI, Spampinato SF, Costantino G, Merlo S, Sortino MA (2021). SIRT1-Dependent Upregulation of BDNF in Human Microglia Challenged with Aβ: An Early but Transient Response Rescued by Melatonin. Biomedicines, 9. 10.3390/biomedicines9050466 PMC8145207 33923297

[143] Ng F, Wijaya L, Tang BL (2015). SIRT1 in the brain-connections with aging-associated disorders and lifespan. Front Cell Neurosci, 9:64. 10.3389/fncel.2015.00064 PMC4353374 25805970

[144] Liao CY, Kennedy BK (2016). SIRT6, oxidative stress, and aging. Cell Res, 26:143-144. 10.1038/cr.2016.8 PMC4746614 26780861

[145] Liao CY, Kennedy BK (2012). Will the real aging Sirtuin please stand up? Cell Res, 22:1215-1217. 10.1038/cr.2012.62 PMC3411170 22508266

[146] Simon M, Yang J, Gigas J, Earley EJ, Hillpot E, Zhang L, et al. (2022). A rare human centenarian variant of SIRT6 enhances genome stability and interaction with Lamin A. Embo j, 41:e110393. 10.15252/embj.2021110393 PMC9627671 36215696

[147] Tennen RI, Chua KF (2011). Chromatin regulation and genome maintenance by mammalian SIRT6. Trends Biochem Sci, 36:39-46. 10.1016/j.tibs.2010.07.009 PMC2991557 20729089

[148] Gredilla R, Sanz A, Lopez-Torres M, Barja G (2001). Caloric restriction decreases mitochondrial free radical generation at complex I and lowers oxidative damage to mitochondrial DNA in the rat heart. Faseb j, 15:1589-1591. 10.1096/fj.00-0764fje 11427495

[149] Bosch-Sierra N, Grau-Del Valle C, Salom C, Zaragoza-Villena B, Perea-Galera L, Falcón-Tapiador R, et al. (2024). Effect of a Very Low-Calorie Diet on Oxidative Stress, Inflammatory and Metabolomic Profile in Metabolically Healthy and Unhealthy Obese Subjects. Antioxidants(Basel), 13. 10.3390/antiox13030302 PMC10967635 38539836

[150] Sohal RS, Weindruch R (1996). Oxidative stress, caloric restriction, and aging. Science, 273:59-63. 10.1126/science.273.5271.59 PMC2987625 8658196

[151] Seidler K, Barrow M (2022). Intermittent fasting and cognitive performance - Targeting BDNF as potential strategy to optimise brain health. Front Neuroendocrinol, 65:100971. 10.1016/j.yfrne.2021.100971 34929259

[152] Bartman S, Coppotelli G, Ross JM (2024). Mitochondrial Dysfunction: A Key Player in Brain Aging and Diseases. Curr Issues Mol Biol, 46:1987-2026. 10.3390/cimb46030130 PMC10969191 38534746

[153] Ayala A, Muñoz MF, Argüelles S (2014). Lipid peroxidation: production, metabolism, and signaling mechanisms of malondialdehyde and 4-hydroxy-2-nonenal. Oxid Med Cell Longev, 2014:360438. 10.1155/2014/360438 PMC4066722 24999379

[154] Kim Y, Zheng X, Ansari Z, Bunnell MC, Herdy JR, Traxler L, et al. (2018). Mitochondrial Aging Defects Emerge in Directly Reprogrammed Human Neurons due to Their Metabolic Profile. Cell Rep, 23:2550-2558. 10.1016/j.celrep.2018.04.105 PMC6478017 29847787

[155] Ahuja P, Ng CF, Pang BPS, Chan WS, Tse MCL, Bi X, et al. (2022). Muscle-generated BDNF (brain derived neurotrophic factor) maintains mitochondrial quality control in female mice. Autophagy, 18:1367-1384. 10.1080/15548627.2021.1985257 PMC9225428 34689722

[156] Swain M, S KS, Tapia K, Dagda RY, Dagda RK (2023). Brain-derived neurotrophic factor protects neurons by stimulating mitochondrial function through protein kinase A. J Neurochem, 167:104-125. 10.1111/jnc.15945 PMC10543477 37688457

[157] Dreher JC, Meyer-Lindenberg A, Kohn P, Berman KF (2008). Age-related changes in midbrain dopaminergic regulation of the human reward system. Proc Natl Acad Sci U S A, 105:15106-15111. 10.1073/pnas.0802127105 PMC2567500 18794529

[158] Kumar JS, Mann JJ (2014). PET tracers for serotonin receptors and their applications. Cent Nerv Syst Agents Med Chem, 14:96-112. 10.2174/1871524914666141030124316 PMC4330993 25360773

[159] Sabandal PR, Saldes EB, Han K-A (2022). Acetylcholine deficit causes dysfunctional inhibitory control in an aging-dependent manner. Scientific Reports, 12:20903. 10.1038/s41598-022-25402-z PMC9719532 36463374

[160] Lee J, Kim HJ (2022). Normal Aging Induces Changes in the Brain and Neurodegeneration Progress: Review of the Structural, Biochemical, Metabolic, Cellular, and Molecular Changes. Front Aging Neurosci, 14:931536. 10.3389/fnagi.2022.931536 PMC9281621 35847660

[161] Armada-Moreira A, Gomes JI, Pina CC, Savchak OK, Gonçalves-Ribeiro J, Rei N, et al. (2020). Going the Extra (Synaptic) Mile: Excitotoxicity as the Road Toward Neurodegenerative Diseases. Front Cell Neurosci, 14:90. 10.3389/fncel.2020.00090 PMC7194075 32390802

[162] Verma M, Lizama BN, Chu CT (2022). Excitotoxicity, calcium and mitochondria: a triad in synaptic neurodegeneration. Transl Neurodegener, 11:3. 10.1186/s40035-021-00278-7 PMC8788129 35078537

[163] Martin JL, Finsterwald C (2011). Cooperation between BDNF and glutamate in the regulation of synaptic transmission and neuronal development. Commun Integr Biol, 4:14-16. 10.4161/cib.4.1.13761 PMC3073261 21509169

[164] Faraji J, Lotfi H, Moharrerie A, Jafari SY, Soltanpour N, Tamannaiee R, et al. (2022). Regional Differences in BDNF Expression and Behavior as a Function of Sex and Enrichment Type: Oxytocin Matters. Cereb Cortex, 32:2985-2999. 10.1093/cercor/bhab395 35059698

[165] Buss EW, Corbett NJ, Roberts JG, Ybarra N, Musial TF, Simkin D, et al. (2021). Cognitive aging is associated with redistribution of synaptic weights in the hippocampus. Proc Natl Acad Sci U S A, 118. 10.1073/pnas.1921481118 PMC7923642 33593893

[166] Wu Z, Chen C, Kang SS, Liu X, Gu X, Yu SP, et al. (2021). Neurotrophic signaling deficiency exacerbates environmental risks for Alzheimer's disease pathogenesis. Proc Natl Acad Sci U S A, 118. 10.1073/pnas.2100986118 PMC8237621 34140411

[167] Oh H, Lewis DA, Sibille E (2016). The Role of BDNF in Age-Dependent Changes of Excitatory and Inhibitory Synaptic Markers in the Human Prefrontal Cortex. Neuropsychopharmacology, 41:3080-3091. 10.1038/npp.2016.126 PMC5101556 27417517

[168] Bimbi G, Tongiorgi E (2024). Chemical LTP induces confinement of BDNF mRNA under dendritic spines and BDNF protein accumulation inside the spines. Front Mol Neurosci, 17:1348445. 10.3389/fnmol.2024.1348445 PMC10914971 38450041

[169] Zagrebelsky M, Tacke C, Korte M (2020). BDNF signaling during the lifetime of dendritic spines. Cell Tissue Res, 382:185-199. 10.1007/s00441-020-03226-5 PMC7529616 32537724

[170] Rex CS, Lauterborn JC, Lin CY, Kramár EA, Rogers GA, Gall CM, et al. (2006). Restoration of long-term potentiation in middle-aged hippocampus after induction of brain-derived neurotrophic factor. J Neurophysiol, 96:677-685. 10.1152/jn.00336.2006 PMC1554892 16707719

[171] Wu HY, Huang CM, Hsu AL, Chen CN, Wu CW, Chen JH (2024). Functional neuroplasticity of facilitation and interference effects on inhibitory control following 3-month physical exercise in aging. Sci Rep, 14:3682. 10.1038/s41598-024-53974-5 PMC10866924 38355770

[172] Bliss TV, Lomo T (1973). Long-lasting potentiation of synaptic transmission in the dentate area of the anaesthetized rabbit following stimulation of the perforant path. J Physiol, 232:331-356. 10.1113/jphysiol.1973.sp010273 PMC1350458 4727084

[173] Lüscher C, Malenka RC (2012). NMDA receptor-dependent long-term potentiation and long-term depression (LTP/LTD). Cold Spring Harb Perspect Biol, 4. 10.1101/cshperspect.a005710 PMC3367554 22510460

[174] Lisman J, Yasuda R, Raghavachari S (2012). Mechanisms of CaMKII action in long-term potentiation. Nat Rev Neurosci, 13:169-182. 10.1038/nrn3192 PMC4050655 22334212

[175] West AE, Chen WG, Dalva MB, Dolmetsch RE, Kornhauser JM, Shaywitz AJ, et al. (2001). Calcium regulation of neuronal gene expression. Proc Natl Acad Sci U S A, 98:11024-11031. 10.1073/pnas.191352298 PMC58677 11572963

[176] Wong LW, Chong YS, Lin W, Kisiswa L, Sim E, Ibáñez CF, et al. (2021). Age-related changes in hippocampal-dependent synaptic plasticity and memory mediated by p75 neurotrophin receptor. Aging Cell, 20:e13305. 10.1111/acel.13305 PMC7884039 33448137

[177] Chou SM, Yen YH, Yuan F, Zhang SC, Chong CM (2023). Neuronal Senescence in the Aged Brain. Aging Dis, 14:1618-1632. 10.14336/AD.2023.0214 PMC10529744 37196117

[178] Ying SW, Futter M, Rosenblum K, Webber MJ, Hunt SP, Bliss TV, et al. (2002). Brain-derived neurotrophic factor induces long-term potentiation in intact adult hippocampus: requirement for ERK activation coupled to CREB and upregulation of Arc synthesis. J Neurosci, 22:1532-1540. 10.1523/JNEUROSCI.22-05-01532.2002 PMC6758896 11880483

[179] Su B, Ji YS, Sun XL, Liu XH, Chen ZY (2014). Brain-derived neurotrophic factor (BDNF)-induced mitochondrial motility arrest and presynaptic docking contribute to BDNF-enhanced synaptic transmission. J Biol Chem, 289:1213-1226. 10.1074/jbc.M113.526129 PMC3894308 24302729

[180] Altman J.2011. The Discovery of Adult Mammalian Neurogenesis. In Neurogenesis in the Adult Brain I: Neurobiology. Seki T., Sawamoto K., Parent J.M., and Alvarez-Buylla A., editors. Tokyo: Springer Japan. 3-46.

[181] Culig L, Chu X, Bohr VA (2022). Neurogenesis in aging and age-related neurodegenerative diseases. Ageing Res Rev, 78:101636. 10.1016/j.arr.2022.101636 PMC9168971 35490966

[182] Babcock KR, Page JS, Fallon JR, Webb AE (2021). Adult Hippocampal Neurogenesis in Aging and Alzheimer's Disease. Stem Cell Reports, 16:681-693. 10.1016/j.stemcr.2021.01.019 PMC8072031 33636114

[183] Schäbitz WR, Steigleder T, Cooper-Kuhn CM, Schwab S, Sommer C, Schneider A, et al. (2007). Intravenous brain-derived neurotrophic factor enhances poststroke sensorimotor recovery and stimulates neurogenesis. Stroke, 38:2165-2172. 10.1161/STROKEAHA.106.477331 17510456

[184] De Vincenti AP, Ríos AS, Paratcha G, Ledda F (2019). Mechanisms That Modulate and Diversify BDNF Functions: Implications for Hippocampal Synaptic Plasticity. Front Cell Neurosci, 13:135. 10.3389/fncel.2019.00135 PMC6465932 31024262

[185] Lupo G (2023). Adult neurogenesis and aging mechanisms: a collection of insights. Sci Rep, 13:18104. 10.1038/s41598-023-45452-1 PMC10593941 37872391

[186] Quaresima S, Istiaq A, Jono H, Cacci E, Ohta K, Lupo G (2022). Assessing the Role of Ependymal and Vascular Cells as Sources of Extracellular Cues Regulating the Mouse Ventricular-Subventricular Zone Neurogenic Niche. Front Cell Dev Biol, 10:845567. 10.3389/fcell.2022.845567 PMC9016221 35450289

[187] Erickson KI, Prakash RS, Voss MW, Chaddock L, Heo S, McLaren M, et al. (2010). Brain-derived neurotrophic factor is associated with age-related decline in hippocampal volume. J Neurosci, 30:5368-5375. 10.1523/JNEUROSCI.6251-09.2010 PMC3069644 20392958

[188] Erickson KI, Voss MW, Prakash RS, Basak C, Szabo A, Chaddock L, et al. (2011). Exercise training increases size of hippocampus and improves memory. Proc Natl Acad Sci U S A, 108:3017-3022. 10.1073/pnas.1015950108 PMC3041121 21282661

[189] Heins N, Malatesta P, Cecconi F, Nakafuku M, Tucker KL, Hack MA, et al. (2002). Glial cells generate neurons: the role of the transcription factor Pax6. Nat Neurosci, 5:308-315. 10.1038/nn828 11896398

[190] Liang S, Zhou J, Yu X, Lu S, Liu R (2024). Neuronal conversion from glia to replenish the lost neurons. Neural Regen Res, 19:1446-1453. 10.4103/1673-5374.386400 PMC10883502 38051886

[191] Niu W, Zang T, Smith DK, Vue TY, Zou Y, Bachoo R, et al. (2015). SOX2 reprograms resident astrocytes into neural progenitors in the adult brain. Stem Cell Reports, 4:780-794. 10.1016/j.stemcr.2015.03.006 PMC4437485 25921813

[192] Metzler MJ, Saucier DM, Metz GA (2013). Enriched childhood experiences moderate age-related motor and cognitive decline. Front Behav Neurosci, 7:1. 10.3389/fnbeh.2013.00001 PMC3575034 23423702

[193] McCreary JK, Metz GAS (2016). Environmental enrichment as an intervention for adverse health outcomes of prenatal stress. Environ Epigenet, 2:dvw013. 10.1093/eep/dvw013 PMC5804528 29492294

[194] Vilar M, Mira H (2016). Regulation of Neurogenesis by Neurotrophins during Adulthood: Expected and Unexpected Roles. Front Neurosci, 10:26. 10.3389/fnins.2016.00026 PMC4746328 26903794

[195] Wu SY, Pan BS, Tsai SF, Chiang YT, Huang BM, Mo FE, et al. (2020). BDNF reverses aging-related microglial activation. J Neuroinflammation, 17:210. 10.1186/s12974-020-01887-1 PMC7362451 32664974

[196] Numakawa T, Odaka H (2022). The Role of Neurotrophin Signaling in Age-Related Cognitive Decline and Cognitive Diseases. Int J Mol Sci, 23. 10.3390/ijms23147726 PMC9320180 35887075

[197] Minichiello L (2009). TrkB signalling pathways in LTP and learning. Nat Rev Neurosci, 10:850-860. 10.1038/nrn2738 19927149

[198] Schirò G, Iacono S, Ragonese P, Aridon P, Salemi G, Balistreri CR (2022). A Brief Overview on BDNF-Trk Pathway in the Nervous System: A Potential Biomarker or Possible Target in Treatment of Multiple Sclerosis? Front Neurol, 13:917527. 10.3389/fneur.2022.917527 PMC9332890 35911894

[199] Chen YR, Li YH, Hsieh TC, Wang CM, Cheng KC, Wang L, et al. (2019). Aging-induced Akt activation involves in aging-related pathologies and Aβ-induced toxicity. Aging Cell, 18:e12989. 10.1111/acel.12989 PMC6612704 31183966

[200] Liu Y, Liu Q, Zhang Z, Yang Y, Zhou Y, Yan H, et al. (2023). The regulatory role of PI3K in ageing-related diseases. Ageing Res Rev, 88:101963. 10.1016/j.arr.2023.101963 37245633

[201] Hardwick JM, Soane L (2013). Multiple functions of BCL-2 family proteins. Cold Spring Harb Perspect Biol, 5. 10.1101/cshperspect.a008722 PMC3552500 23378584

[202] Oltvai ZN, Milliman CL, Korsmeyer SJ (1993). Bcl-2 heterodimerizes in vivo with a conserved homolog, Bax, that accelerates programmed cell death. Cell, 74:609-619. 10.1016/0092-8674(93)90509-o 8358790

[203] Vitagliano O, Addeo R, D'Angelo V, Indolfi C, Indolfi P, Casale F (2013). The Bcl-2/Bax and Ras/Raf/MEK/ERK signaling pathways: implications in pediatric leukemia pathogenesis and new prospects for therapeutic approaches. Expert Rev Hematol, 6:587-597. 10.1586/17474086.2013.827415 24083449

[204] Schäbitz WR, Sommer C, Zoder W, Kiessling M, Schwaninger M, Schwab S (2000). Intravenous brain-derived neurotrophic factor reduces infarct size and counterregulates Bax and Bcl-2 expression after temporary focal cerebral ischemia. Stroke, 31:2212-2217. 10.1161/01.str.31.9.2212 10978054

[205] Roberts PJ, Der CJ (2007). Targeting the Raf-MEK-ERK mitogen-activated protein kinase cascade for the treatment of cancer. Oncogene, 26:3291-3310. 10.1038/sj.onc.1210422 17496923

[206] Jaaro H, Rubinfeld H, Hanoch T, Seger R (1997). Nuclear translocation of mitogen-activated protein kinase kinase (MEK1) in response to mitogenic stimulation. Proc Natl Acad Sci U S A, 94:3742-3747. 10.1073/pnas.94.8.3742 PMC20511 9108048

[207] Shabestari RM, Safa M, Alikarami F, Banan M, Kazemi A (2017). CREB knockdown inhibits growth and induces apoptosis in human pre-B acute lymphoblastic leukemia cells through inhibition of prosurvival signals. Biomedicine & Pharmacotherapy, 87:274-279. 10.1016/j.biopha.2016.12.070 28063408

[208] Pugazhenthi S, Wang M, Pham S, Sze C-I, Eckman CB (2011). Downregulation of CREB expression in Alzheimer's brain and in Aβ-treated rat hippocampal neurons. Molecular Neurodegeneration, 6:60. 10.1186/1750-1326-6-60 PMC3174124 21854604

[209] Yoo JM, Lee BD, Sok DE, Ma JY, Kim MR (2017). Neuroprotective action of N-acetyl serotonin in oxidative stress-induced apoptosis through the activation of both TrkB/CREB/BDNF pathway and Akt/Nrf2/Antioxidant enzyme in neuronal cells. Redox Biol, 11:592-599. 10.1016/j.redox.2016.12.034 PMC5247570 28110215

[210] Esvald EE, Tuvikene J, Sirp A, Patil S, Bramham CR, Timmusk T (2020). CREB Family Transcription Factors Are Major Mediators of BDNF Transcriptional Autoregulation in Cortical Neurons. J Neurosci, 40:1405-1426. 10.1523/JNEUROSCI.0367-19.2019 PMC7044735 31915257

[211] Zhou J, Du T, Li B, Rong Y, Verkhratsky A, Peng L (2015). Crosstalk Between MAPK/ERK and PI3K/AKT Signal Pathways During Brain Ischemia/Reperfusion. ASN Neuro, 7. 10.1177/1759091415602463 PMC4601130 26442853

[212] Dent P (2014). Crosstalk between ERK, AKT, and cell survival. Cancer Biol Ther, 15:245-246. 10.4161/cbt.27541 PMC3974823 24424114

[213] Li Y, Li F, Qin D, Chen H, Wang J, Wang J, et al. (2022). The role of brain derived neurotrophic factor in central nervous system. Front Aging Neurosci, 14:986443. 10.3389/fnagi.2022.986443 PMC9493475 36158555

[214] Baechle JJ, Chen N, Makhijani P, Winer S, Furman D, Winer DA (2023). Chronic inflammation and the hallmarks of aging. Mol Metab, 74:101755. 10.1016/j.molmet.2023.101755 PMC10359950 37329949

[215] Cribbs DH, Berchtold NC, Perreau V, Coleman PD, Rogers J, Tenner AJ, et al. (2012). Extensive innate immune gene activation accompanies brain aging, increasing vulnerability to cognitive decline and neurodegeneration: a microarray study. J Neuroinflammation, 9:179. 10.1186/1742-2094-9-179 PMC3419089 22824372

[216] Grabert K, Michoel T, Karavolos MH, Clohisey S, Baillie JK, Stevens MP, et al. (2016). Microglial brain region-dependent diversity and selective regional sensitivities to aging. Nat Neurosci, 19:504-516. 10.1038/nn.4222 PMC4768346 26780511

[217] Sierra A, Gottfried-Blackmore AC, McEwen BS, Bulloch K (2007). Microglia derived from aging mice exhibit an altered inflammatory profile. Glia, 55:412-424. 10.1002/glia.20468 17203473

[218] Clarke LE, Liddelow SA, Chakraborty C, Münch AE, Heiman M, Barres BA (2018). Normal aging induces A1-like astrocyte reactivity. Proc Natl Acad Sci U S A, 115:E1896-e1905. 10.1073/pnas.1800165115 PMC5828643 29437957

[219] Ekdahl CT, Claasen JH, Bonde S, Kokaia Z, Lindvall O (2003). Inflammation is detrimental for neurogenesis in adult brain. Proc Natl Acad Sci U S A, 100:13632-13637. 10.1073/pnas.2234031100 PMC263865 14581618

[220] Arvanitaki ES, Goulielmaki E, Gkirtzimanaki K, Niotis G, Tsakani E, Nenedaki E, et al. (2024). Microglia-derived extracellular vesicles trigger age-related neurodegeneration upon DNA damage. Proc Natl Acad Sci U S A, 121:e2317402121. 10.1073/pnas.2317402121 PMC11047102 38635632

[221] Witte AV, Fobker M, Gellner R, Knecht S, Flöel A (2009). Caloric restriction improves memory in elderly humans. Proc Natl Acad Sci U S A, 106:1255-1260. 10.1073/pnas.0808587106 PMC2633586 19171901

[222] van Praag H, Shubert T, Zhao C, Gage FH (2005). Exercise enhances learning and hippocampal neurogenesis in aged mice. J Neurosci, 25:8680-8685. 10.1523/JNEUROSCI.1731-05.2005 PMC1360197 16177036

[223] Parkhurst CN, Yang G, Ninan I, Savas JN, Yates JR 3rd, Lafaille JJ, et al. (2013). Microglia promote learning-dependent synapse formation through brain-derived neurotrophic factor. Cell, 155:1596-1609. 10.1016/j.cell.2013.11.030 PMC4033691 24360280

[224] Prowse N, Hayley S (2021). Microglia and BDNF at the crossroads of stressor related disorders: Towards a unique trophic phenotype. Neurosci Biobehav Rev, 131:135-163. 10.1016/j.neubiorev.2021.09.018 34537262

[225] Jiang Y, Wei N, Lu T, Zhu J, Xu G, Liu X (2011). Intranasal brain-derived neurotrophic factor protects brain from ischemic insult via modulating local inflammation in rats. Neuroscience, 172:398-405. 10.1016/j.neuroscience.2010.10.054 21034794

[226] Makar TK, Trisler D, Sura KT, Sultana S, Patel N, Bever CT (2008). Brain derived neurotrophic factor treatment reduces inflammation and apoptosis in experimental allergic encephalomyelitis. J Neurol Sci, 270:70-76. 10.1016/j.jns.2008.02.011 18374360

[227] Bovolenta R, Zucchini S, Paradiso B, Rodi D, Merigo F, Navarro Mora G, et al. (2010). Hippocampal FGF-2 and BDNF overexpression attenuates epileptogenesis-associated neuroinflammation and reduces spontaneous recurrent seizures. J Neuroinflammation, 7:81. 10.1186/1742-2094-7-81 PMC2993685 21087489

[228] Charlton T, Prowse N, McFee A, Heiratifar N, Fortin T, Paquette C, et al. (2023). Brain-derived neurotrophic factor (BDNF) has direct anti-inflammatory effects on microglia. Front Cell Neurosci, 17:1188672. 10.3389/fncel.2023.1188672 PMC10315457 37404293

[229] Papathanassoglou ED, Miltiadous P, Karanikola MN (2015). May BDNF Be Implicated in the Exercise-Mediated Regulation of Inflammation? Critical Review and Synthesis of Evidence. Biol Res Nurs, 17:521-539. 10.1177/1099800414555411 25358684

[230] Xu D, Lian D, Wu J, Liu Y, Zhu M, Sun J, et al. (2017). Brain-derived neurotrophic factor reduces inflammation and hippocampal apoptosis in experimental Streptococcus pneumoniae meningitis. J Neuroinflammation, 14:156. 10.1186/s12974-017-0930-6 PMC5545027 28778220

[231] Balan I, Grusca A, O'Buckley TK, Morrow AL (2023). Neurosteroid [3α,5α]-3-hydroxy-pregnan-20-one enhances IL-10 production via endosomal TRIF-dependent TLR4 signaling pathway. Front Endocrinol (Lausanne), 14:1299420. 10.3389/fendo.2023.1299420 PMC10765172 38179300

[232] Xiong X, Zeng M, Peng X, Feng C, Li C, Weng W, et al. (2023). Serum brain-derived neurotrophic factor (BDNF) as predictors of childhood neuroblastoma relapse. BMC Cancer, 23:670. 10.1186/s12885-023-11159-9 PMC10351183 37460933

[233] Honey D, Wosnitzka E, Klann E, Weinhard L (2022). Analysis of microglial BDNF function and expression in the motor cortex. Front Cell Neurosci, 16:961276. 10.3389/fncel.2022.961276 PMC9885322 36726454

[234] Komori T, Okamura K, Ikehara M, Yamamuro K, Endo N, Okumura K, et al. (2024). Brain-derived neurotrophic factor from microglia regulates neuronal development in the medial prefrontal cortex and its associated social behavior. Mol Psychiatry. 10.1038/s41380-024-02413-y PMC11189755 38243072

[235] Andjelkovic AV, Situ M, Citalan-Madrid AF, Stamatovic SM, Xiang J, Keep RF (2023). Blood-Brain Barrier Dysfunction in Normal Aging and Neurodegeneration: Mechanisms, Impact, and Treatments. Stroke, 54:661-672. 10.1161/STROKEAHA.122.040578 PMC9993074 36848419

[236] Banks WA, Reed MJ, Logsdon AF, Rhea EM, Erickson MA (2021). Healthy aging and the blood-brain barrier. Nat Aging, 1:243-254. 10.1038/s43587-021-00043-5 PMC8340949 34368785

[237] Goodall EF, Wang C, Simpson JE, Baker DJ, Drew DR, Heath PR, et al. (2018). Age-associated changes in the blood-brain barrier: comparative studies in human and mouse. Neuropathol Appl Neurobiol, 44:328-340. 10.1111/nan.12408 PMC5900918 28453876

[238] Hafezi-Moghadam A, Thomas KL, Wagner DD (2007). ApoE deficiency leads to a progressive age-dependent blood-brain barrier leakage. Am J Physiol Cell Physiol, 292:C1256-1262. 10.1152/ajpcell.00563.2005 16870825

[239] Lee P, Kim J, Williams R, Sandhir R, Gregory E, Brooks WM, et al. (2012). Effects of aging on blood brain barrier and matrix metalloproteases following controlled cortical impact in mice. Exp Neurol, 234:50-61. 10.1016/j.expneurol.2011.12.016 PMC4042317 22201549

[240] Balietti M, Giuli C, Conti F (2018). Peripheral Blood Brain-Derived Neurotrophic Factor as a Biomarker of Alzheimer's Disease: Are There Methodological Biases? Mol Neurobiol, 55:6661-6672. 10.1007/s12035-017-0866-y PMC6061178 29330839

[241] Selvakumar K, Bavithra S, Krishnamoorthy G, Arunakaran J (2018). Impact of quercetin on tight junctional proteins and BDNF signaling molecules in hippocampus of PCBs-exposed rats. Interdiscip Toxicol, 11:294-305. 10.2478/intox-2018-0029 PMC6853011 31762681

[242] Greene C, Connolly R, Brennan D, Laffan A, O'Keeffe E, Zaporojan L, et al. (2024). Blood-brain barrier disruption and sustained systemic inflammation in individuals with long COVID-associated cognitive impairment. Nat Neurosci, 27:421-432. 10.1038/s41593-024-01576-9 PMC10917679 38388736

[243] Elahy M, Jackaman C, Mamo JC, Lam V, Dhaliwal SS, Giles C, et al. (2015). Blood-brain barrier dysfunction developed during normal aging is associated with inflammation and loss of tight junctions but not with leukocyte recruitment. Immun Ageing, 12:2. 10.1186/s12979-015-0029-9 PMC4362825 25784952

[244] Barros LF, San Martín A, Ruminot I, Sandoval PY, Fernández-Moncada I, Baeza-Lehnert F, et al. (2017). Near-critical GLUT1 and Neurodegeneration. J Neurosci Res, 95:2267-2274. 10.1002/jnr.23998 28150866

[245] Winkler EA, Nishida Y, Sagare AP, Rege SV, Bell RD, Perlmutter D, et al. (2015). GLUT1 reductions exacerbate Alzheimer's disease vasculo-neuronal dysfunction and degeneration. Nat Neurosci, 18:521-530. 10.1038/nn.3966 PMC4734893 25730668

[246] Vulturar R, Chiș A, Pintilie S, Farcaș IM, Botezatu A, Login CC, et al. (2022). One Molecule for Mental Nourishment and More: Glucose Transporter Type 1-Biology and Deficiency Syndrome. Biomedicines, 10. 10.3390/biomedicines10061249 PMC9219734 35740271

[247] Xu L, Liu R, Qin Y, Wang T (2023). Brain metabolism in Alzheimer's disease: biological mechanisms of exercise. Transl Neurodegener, 12:33. 10.1186/s40035-023-00364-y PMC10294518 37365651

[248] Tang M, Park SH, Petri S, Yu H, Rueda CB, Abel ED, et al. (2021). An early endothelial cell-specific requirement for Glut1 is revealed in Glut1 deficiency syndrome model mice. JCI Insight, 6. 10.1172/jci.insight.145789 PMC7934852 33351789

[249] Wang L, Pavlou S, Du X, Bhuckory M, Xu H, Chen M (2019). Glucose transporter 1 critically controls microglial activation through facilitating glycolysis. Mol Neurodegener, 14:2. 10.1186/s13024-019-0305-9 PMC6329071 30634998

[250] Tyrrell DJ, Blin MG, Song J, Wood SC, Goldstein DR (2020). Aging Impairs Mitochondrial Function and Mitophagy and Elevates Interleukin 6 Within the Cerebral Vasculature. J Am Heart Assoc, 9:e017820. 10.1161/JAHA.120.017820 PMC7763766 33225820

[251] Surnar B, Basu U, Banik B, Ahmad A, Marples B, Kolishetti N, et al. (2018). Nanotechnology-mediated crossing of two impermeable membranes to modulate the stars of the neurovascular unit for neuroprotection. Proc Natl Acad Sci U S A, 115:E12333-e12342. 10.1073/pnas.1816429115 PMC6310851 30530697

[252] Markham A, Bains R, Franklin P, Spedding M (2014). Changes in mitochondrial function are pivotal in neurodegenerative and psychiatric disorders: how important is BDNF? Br J Pharmacol, 171:2206-2229. 10.1111/bph.12531 PMC3976631 24720259

[253] Wang X, Su B, Lee HG, Li X, Perry G, Smith MA, et al. (2009). Impaired balance of mitochondrial fission and fusion in Alzheimer's disease. J Neurosci, 29:9090-9103. 10.1523/JNEUROSCI.1357-09.2009 PMC2735241 19605646

[254] Grimm A, Eckert A (2017). Brain aging and neurodegeneration: from a mitochondrial point of view. J Neurochem, 143:418-431. 10.1111/jnc.14037 PMC5724505 28397282

[255] Bondy SC (2024). Mitochondrial Dysfunction as the Major Basis of Brain Aging. Biomolecules, 14. 10.3390/biom14040402 PMC11048299 38672420

[256] Marques F, Sousa JC, Sousa N, Palha JA (2013). Blood-brain-barriers in aging and in Alzheimer's disease. Mol Neurodegener, 8:38. 10.1186/1750-1326-8-38 PMC4015275 24148264

[257] Gejl AK, Enevold C, Bugge A, Andersen MS, Nielsen CH, Andersen LB (2019). Associations between serum and plasma brain-derived neurotrophic factor and influence of storage time and centrifugation strategy. Scientific Reports, 9:9655. 10.1038/s41598-019-45976-5 PMC6609657 31273250

[258] Nettiksimmons J, Simonsick EM, Harris T, Satterfield S, Rosano C, Yaffe K (2014). The associations between serum brain-derived neurotrophic factor, potential confounders, and cognitive decline: a longitudinal study. PLoS One, 9:e91339. 10.1371/journal.pone.0091339 PMC3966768 24670553

[259] Polacchini A, Metelli G, Francavilla R, Baj G, Florean M, Mascaretti LG, et al. (2015). A method for reproducible measurements of serum BDNF: comparison of the performance of six commercial assays. Scientific Reports, 5:17989. 10.1038/srep17989 PMC4675070 26656852

[260] Shimada H, Makizako H, Doi T, Yoshida D, Tsutsumimoto K, Anan Y, et al. (2014). A large, cross-sectional observational study of serum BDNF, cognitive function, and mild cognitive impairment in the elderly. Front Aging Neurosci, 6:69. 10.3389/fnagi.2014.00069 PMC3995061 24782766

[261] Woolley JD, Strobl EV, Shelly WB, Karydas AM, Robin Ketelle RN, Wolkowitz OM, et al. (2012). BDNF serum concentrations show no relationship with diagnostic group or medication status in neurodegenerative disease. Curr Alzheimer Res, 9:815-821. 10.2174/156720512802455395 PMC3176995 21605064

[262] Pfefferbaum A, Sullivan EV (2015). Cross-sectional versus longitudinal estimates of age-related changes in the adult brain: overlaps and discrepancies. Neurobiol Aging, 36:2563-2567. 10.1016/j.neurobiolaging.2015.05.005 PMC4523414 26059713

[263] Vyas C, Mischoulon D, Reynolds C, Cook N, Rifai N, Mora S, et al. (2023). Cross-sectional and longitudinal associations between serum BDNF and late-life depression and exploration of the role of serum BDNF in a study of vitamin D3 and omega-3 supplements for late-life depression prevention. The American Journal of Geriatric Psychiatry, 31:e32-e33.

[264] Zhang X, Meirelles OD, Li Z, Yaffe K, Bryan RN, Qiu C, et al. (2023). Sedentary behavior, brain-derived neurotrophic factor and brain structure in midlife: A longitudinal brain MRI sub-study of the coronary artery risk development in young adults study. Front Dement, 2:1110553. 10.3389/frdem.2023.1110553 PMC11285629 39081995

[265] Knüsel B, Beck KD, Winslow JW, Rosenthal A, Burton LE, Widmer HR, et al. (1992). Brain-derived neurotrophic factor administration protects basal forebrain cholinergic but not nigral dopaminergic neurons from degenerative changes after axotomy in the adult rat brain. J Neurosci, 12:4391-4402. 10.1523/JNEUROSCI.12-11-04391.1992 PMC6576000 1432101

[266] Klein AB, Williamson R, Santini MA, Clemmensen C, Ettrup A, Rios M, et al. (2011). Blood BDNF concentrations reflect brain-tissue BDNF levels across species. Int J Neuropsychopharmacol, 14:347-353. 10.1017/S1461145710000738 20604989

[267] Wang Q, Zhang L, Yuan X, Ou Y, Zhu X, Cheng Z, et al. (2016). The Relationship between the Bcl-2/Bax Proteins and the Mitochondria-Mediated Apoptosis Pathway in the Differentiation of Adipose-Derived Stromal Cells into Neurons. PLoS One, 11:e0163327. 10.1371/journal.pone.0163327 PMC5051896 27706181

[268] Schroer J, Warm D, De Rosa F, Luhmann HJ, Sinning A (2023). Activity-dependent regulation of the BAX/BCL-2 pathway protects cortical neurons from apoptotic death during early development. Cell Mol Life Sci, 80:175. 10.1007/s00018-023-04824-6 PMC10239391 37269320

[269] De Gioia R, Biella F, Citterio G, Rizzo F, Abati E, Nizzardo M, et al. (2020). Neural Stem Cell Transplantation for Neurodegenerative Diseases. Int J Mol Sci, 21. 10.3390/ijms21093103 PMC7247151 32354178

[270] Hoover TDM, Gerlinde AS. (2024 (in press)). What Comes after Moral Injury? - Considerations of Post-Traumatic Growth. Trauma Care, 4.

[271] Duman RS, Li N (2012). A neurotrophic hypothesis of depression: role of synaptogenesis in the actions of NMDA receptor antagonists. Philos Trans R Soc Lond B Biol Sci, 367:2475-2484. 10.1098/rstb.2011.0357 PMC3405673 22826346

[272] Arosio B, Guerini FR, Voshaar RCO, Aprahamian I (2021). Blood Brain-Derived Neurotrophic Factor (BDNF) and Major Depression: Do We Have a Translational Perspective? Front Behav Neurosci, 15:626906. 10.3389/fnbeh.2021.626906 PMC7906965 33643008

[273] Jaggar M, Fanibunda SE, Ghosh S, Duman RS, Vaidya VA. 2019. Chapter 6 - The Neurotrophic Hypothesis of Depression Revisited: New Insights and Therapeutic Implications. In Neurobiology of Depression. Quevedo J., Carvalho A.F., and Zarate C.A., editors: Academic Press. 43-62.

[274] Mahncke HW, Connor BB, Appelman J, Ahsanuddin ON, Hardy JL, Wood RA, et al. (2006). Memory enhancement in healthy older adults using a brain plasticity-based training program: a randomized, controlled study. Proc Natl Acad Sci U S A, 103:12523-12528. 10.1073/pnas.0605194103 PMC1526649 16888038

[275] Cabral DF, Hinchman CA, Nunez C, Rice J, Loewenstein DA, Cahalin LP, et al. (2021). Harnessing Neuroplasticity to Promote Brain Health in Aging Adults: Protocol for the MOVE-Cog Intervention Study. JMIR Res Protoc, 10:e33589. 10.2196/33589 PMC8663452 34817393

[276] Kase Y, Shimazaki T, Okano H (2020). Current understanding of adult neurogenesis in the mammalian brain: how does adult neurogenesis decrease with age? Inflamm Regen, 40:10. 10.1186/s41232-020-00122-x PMC7302355 32566044

[277] Antonijevic M, Dallemagne P, Rochais C (2024). Inducing neuronal regeneration and differentiation via the BDNF/TrkB signaling pathway: a key target against neurodegenerative diseases? Neural Regen Res, 19:495-496. 10.4103/1673-5374.380896 PMC10581589 37721270

[278] Matrone C, Ciotti MT, Mercanti D, Marolda R, Calissano P (2008). NGF and BDNF signaling control amyloidogenic route and Abeta production in hippocampal neurons. Proc Natl Acad Sci U S A, 105:13139-13144. 10.1073/pnas.0806133105 PMC2525562 18728191

[279] Ibrahim AM, Chauhan L, Bhardwaj A, Sharma A, Fayaz F, Kumar B, et al. (2022). Brain-Derived Neurotropic Factor in Neurodegenerative Disorders. Biomedicines, 10. 10.3390/biomedicines10051143 PMC9138678 35625880

[280] Lautrup S, Myrup Holst C, Yde A, Asmussen S, Thinggaard V, Larsen K, et al. (2023). The role of aging and brain-derived neurotrophic factor signaling in expression of base excision repair genes in the human brain. Aging Cell, 22:e13905. 10.1111/acel.13905 PMC10497833 37334527

[281] Pisani A, Paciello F, Del Vecchio V, Malesci R, De Corso E, Cantone E, et al. (2023). The Role of BDNF as a Biomarker in Cognitive and Sensory Neurodegeneration. J Pers Med, 13. 10.3390/jpm13040652 PMC10140880 37109038

[282] Chen A, Chen X, Deng J, Wei J, Qian H, Huang Y, et al. (2022). Dexmedetomidine alleviates olfactory cognitive dysfunction by promoting neurogenesis in the subventricular zone of hypoxic-ischemic neonatal rats. Front Pharmacol, 13:983920. 10.3389/fphar.2022.983920 PMC9437207 36059991

[283] Wendimu MY, Hooks SB (2022). Microglia Phenotypes in Aging and Neurodegenerative Diseases. Cells, 11. 10.3390/cells11132091 PMC9266143 35805174

[284] Wu CC, Lien CC, Hou WH, Chiang PM, Tsai KJ (2016). Gain of BDNF Function in Engrafted Neural Stem Cells Promotes the Therapeutic Potential for Alzheimer's Disease. Sci Rep, 6:27358. 10.1038/srep27358 PMC4893631 27264956

[285] Bathina S, Das UN (2015). Brain-derived neurotrophic factor and its clinical implications. Arch Med Sci, 11:1164-1178. 10.5114/aoms.2015.56342 PMC4697050 26788077

[286] Mattson MP, Maudsley S, Martin B (2004). BDNF and 5-HT: a dynamic duo in age-related neuronal plasticity and neurodegenerative disorders. Trends Neurosci, 27:589-594. 10.1016/j.tins.2004.08.001 15374669

[287] Marosi K, Mattson MP (2014). BDNF mediates adaptive brain and body responses to energetic challenges. Trends Endocrinol Metab, 25:89-98. 10.1016/j.tem.2013.10.006 PMC3915771 24361004

[288] Kandimalla R, Thirumala V, Reddy PH (2017). Is Alzheimer's disease a Type 3 Diabetes? A critical appraisal. Biochim Biophys Acta Mol Basis Dis, 1863:1078-1089. 10.1016/j.bbadis.2016.08.018 PMC5344773 27567931

[289] Rumajogee P, Vergé D, Hanoun N, Brisorgueil MJ, Hen R, Lesch KP, et al. (2004). Adaption of the serotoninergic neuronal phenotype in the absence of 5-HT autoreceptors or the 5-HT transporter: involvement of BDNF and cAMP. Eur J Neurosci, 19:937-944. 10.1111/j.0953-816x.2004.03194.x 15009141

[290] Leschik J, Gentile A, Cicek C, Péron S, Tevosian M, Beer A, et al. (2022). Brain-derived neurotrophic factor expression in serotonergic neurons improves stress resilience and promotes adult hippocampal neurogenesis. Prog Neurobiol, 217:102333. 10.1016/j.pneurobio.2022.102333 35872219

[291] Bakos J, Srancikova A, Havranek T, Bacova Z (2018). Molecular Mechanisms of Oxytocin Signaling at the Synaptic Connection. Neural Plast, 2018:4864107. 10.1155/2018/4864107 PMC6051047 30057594

[292] Bakoyiannis I, Daskalopoulou A, Pergialiotis V, Perrea D (2019). Phytochemicals and cognitive health: Are flavonoids doing the trick? Biomed Pharmacother, 109:1488-1497. 10.1016/j.biopha.2018.10.086 30551400

[293] Neshatdoust S, Saunders C, Castle SM, Vauzour D, Williams C, Butler L, et al. (2016). High-flavonoid intake induces cognitive improvements linked to changes in serum brain-derived neurotrophic factor: Two randomised, controlled trials. Nutr Healthy Aging, 4:81-93. 10.3233/NHA-1615 PMC5166520 28035345

[294] Morton L, Paton C, Braakhuis A (2024). The Effects of Polyphenol Supplementation on BDNF, Cytokines and Cognition in Trained Male Cyclists following Acute Ozone Exposure during High-Intensity Cycling. Nutrients, 16. 10.3390/nu16020233 PMC10819340 38257125

[295] Wrann CD, White JP, Salogiannnis J, Laznik-Bogoslavski D, Wu J, Ma D, et al. (2013). Exercise induces hippocampal BDNF through a PGC-1α/FNDC5 pathway. Cell Metab, 18:649-659. 10.1016/j.cmet.2013.09.008 PMC3980968 24120943

[296] Colcombe S, Kramer AF (2003). Fitness effects on the cognitive function of older adults: a meta-analytic study. Psychol Sci, 14:125-130. 10.1111/1467-9280.t01-1-01430 12661673

[297] Cotman CW, Berchtold NC, Christie LA (2007). Exercise builds brain health: key roles of growth factor cascades and inflammation. Trends Neurosci, 30:464-472. 10.1016/j.tins.2007.06.011 17765329

[298] Kam TI, Park H, Chou SC, Van Vranken JG, Mittenbühler MJ, Kim H, et al. (2022). Amelioration of pathologic α-synuclein-induced Parkinson's disease by irisin. Proc Natl Acad Sci U S A, 119:e2204835119. 10.1073/pnas.2204835119 PMC9457183 36044549

